# Non-Coding RNAs from Cardiac Development to Disease: Regulatory Context, Regenerative Potential, and Translational Limits

**DOI:** 10.3390/ijms27177793

**Published:** 2026-08-31

**Authors:** Le Wang, Yuyang Zhao, Ruiyang Zhang, Xuan Ning, Bo Jin

**Affiliations:** 1 Department of Laboratory Medicine, Peking University First Hospital, Beijing 100034, China; 2 School of Basic Medical Sciences, Peking University Health Science Center, No. 38 Xueyuan Road, Haidian District, Beijing 100191, China; 18210764332@163.com (Y.Z.);

**Keywords:** non-coding RNA, cardiac development, cardiac regeneration, cardiovascular disease, cardiomyocyte proliferation, extracellular vesicles, RNA therapeutics, circulating biomarkers

## Abstract

Non-coding RNAs (ncRNAs) are often reviewed as lists of molecules associated with individual cardiovascular phenotypes. Here, we instead ask how ncRNAs alter the probability, timing, and stability of cardiovascular cell-state transitions across development, injury, and disease. We organize evidence for microRNAs, long non-coding RNAs, circular RNAs, and extracellular-vesicle-associated RNAs around four regulatory operations: chromatin and transcriptional competence, post-transcriptional tuning, multicellular communication, and persistence or reversibility of the response. Cardiac development provides the reference trajectory from mesoderm specification through progenitor commitment, morphogenesis, and functional maturation. Regeneration is evaluated separately from DNA synthesis or cardioprotection and requires daughter-cardiomyocyte formation, maturation, electrical integration, scar replacement, and durable functional recovery. The disease section uses a bounded cardiovascular scope: atherosclerosis and hypertension are included as determinants of coronary supply and ventricular load, whereas renal and pulmonary examples are restricted to mechanisms that directly affect cardiac afterload or ventricular adaptation. Across these settings, the most recurrent ncRNAs are not universal switches; their effects depend on cell identity, subcellular localization, endogenous abundance, dose, and disease phase. We therefore grade mechanistic claims by target engagement, cell and species specificity, physiological stoichiometry, in vivo rescue, and human validation. This framework converts a molecule-by-molecule catalogue into a critical account of when ncRNAs are causal regulators, context-dependent modifiers, or biomarkers of changing tissue composition.

## 1. Introduction

Cardiovascular health depends on ordered cell-state transitions. During embryogenesis, progenitors acquire myocardial, endocardial, vascular, fibroblast, and neural-crest-derived identities and assemble a mechanically and electrically integrated organ. After injury, the adult heart must preserve surviving cells, restore perfusion, resolve inflammation, reorganize matrix, and—only in restricted settings—replace lost cardiomyocytes. Cardiovascular disease can therefore be viewed partly as failed, incomplete, or maladaptive reuse of regulatory programs that normally coordinate cell identity and tissue organization. A review spanning development, regeneration, and disease is useful only if it explains these shared regulatory principles rather than treating the three settings as parallel catalogues.

The term non-coding RNA (ncRNA) covers mechanistically different entities. MicroRNAs (miRNAs) use sequence-guided post-transcriptional repression; long non-coding RNAs (lncRNAs) may regulate chromatin, transcription, RNA processing, or protein assembly; circular RNAs (circRNAs) can bind proteins or RNAs and, in selected cases, be translated; and extracellular-vesicle-associated RNA denotes a transport context rather than a separate RNA biotype. These labels do not establish mechanism. Endogenous abundance, subcellular localization, accessible binding sites, molecular stoichiometry, isoform identity, and species conservation determine whether a proposed interaction is physiologically plausible [1,2,3].

These context dependencies also create a therapeutic opportunity. An ncRNA can coordinately shift a disease-relevant gene network, while cell-selective delivery, transient exposure, and stage-specific treatment can in principle confine that effect to the compartment and time window in which it is beneficial. Conversely, the same network breadth makes uncontrolled distribution or prolonged dosing hazardous. Defining the RNA, cell, localization, dose, and disease phase is therefore not only a mechanistic exercise but a prerequisite for designing controllable cardiovascular RNA therapeutics and for interpreting the emerging clinical trials summarized below [4,5,6].

The central question of this review is how ncRNAs alter the probability, timing, or stability of a cardiovascular cell state. We use four organizing operations: chromatin and transcriptional competence; post-transcriptional tuning of fate and stress networks; communication among cardiomyocytes, endothelium, fibroblasts, immune cells, and progenitors; and the persistence or reversibility of an RNA response. Individual ncRNAs are used as evidence for these operations, rather than as the organizing units of the review [7,8,9,10,11,12,13,14,15].

Throughout the review, each example is therefore introduced in the same order: the cardiac process at issue, the experimental model, the directly observed result, and only then the inference and its limitation. Intact embryonic perturbation, directed embryonic stem cell (ESC)/induced pluripotent stem cell (iPSC) differentiation, neonatal maturation, and adult injury or stress answer different biological questions and are not treated as interchangeable evidence. This context-first structure is intended to show why an ncRNA is relevant before describing its molecular partners.

This framework also separates outcomes that are frequently conflated. Developmental morphogenesis creates a new organ; repair preserves or restores tissue after injury; cardioprotection limits cell loss; and true myocardial regeneration requires production and integration of new cardiomyocytes. The same RNA circuit may be adaptive during a brief developmental or acute-injury window but maladaptive when activated persistently in the adult heart. Evidence must therefore be interpreted with respect to cell type, developmental or disease phase, dose, and tissue-level endpoint [16,17,18,19,20,21].

At the tissue level, no one RNA or cell population acts alone. Endothelial supply, extracellular matrix, immune resolution, and electrical coupling determine whether a molecular change yields functional recovery. Extracellular-vesicle studies illustrate this multicellular layer, while also showing why cargo identity, delivered copy number, and recipient-cell target engagement must be demonstrated before benefit is attributed to a particular RNA [22,23,24].

Accordingly, this review has a bounded cardiovascular—not exclusively myocardial—scope. Atherosclerosis is included as the principal substrate for coronary ischemia, and hypertension as a determinant of ventricular load and remodeling. Renal mechanisms are considered only where ncRNAs modify the renin–angiotensin–aldosterone axis or blood pressure, and pulmonary arterial hypertension only as a model of vascular remodeling coupled directly to right-ventricular adaptation. The literature is selected when it meets at least one of three criteria: direct relevance to cardiac formation or function, mechanistic continuity with developmental or regenerative programs, or evidence in a defined cardiovascular cell or tissue. The resulting aim is to identify reproducible regulatory principles and to distinguish them from associations, extrapolations, and model-specific narratives.

## 2. ncRNAs in Cardiac Development and Morphogenesis

Cardiogenesis is a sequence of linked biological problems. Gastrulation generates lateral-plate mesoderm; first- and second-heart-field progenitors then acquire regional identities, fuse into a primitive heart tube, and undergo looping, chamber formation, trabeculation, and outflow-tract alignment. Endocardial cells and cardiac neural crest cells contribute to cushions, valves, septation, and great-vessel patterning, while epicardial derivatives and fibroblasts organize the matrix and coronary support. Cardiomyocytes subsequently mature their sarcomeres, metabolism, calcium handling, and conduction. ncRNAs are relevant when they alter one of these defined transitions, not simply because their abundance changes during embryogenesis [1,7,8,9,10,11,12,13,25,26,27,28,29,30,31,32,33].

This section is organized by developmental question and cell context. Section 2.1 addresses acquisition of cardiac competence and lineage commitment; Section 2.2 separates early cardiomyocyte differentiation from postnatal maturation and adult stress; Section 2.3 considers endocardial and endothelial morphogenesis; Section 2.4 identifies the evidence gap in developmental fibroblast and matrix regulation; and Section 2.5 examines neural-crest survival and outflow-tract septation. In each case, the developmental phenotype is stated before the ncRNA example [29,30,31,32,33].

A cell-resolved interpretation is essential. Whole-heart RNA changes may reflect altered proportions of cardiomyocytes, fibroblasts, endothelial cells, vascular smooth-muscle cells, neural-crest derivatives, or immune cells rather than regulation within one lineage. Sorted-cell, single-cell, spatial, or lineage-restricted perturbation data therefore carry more mechanistic weight than bulk expression alone. Species also matter: many lncRNAs lack primary-sequence conservation, and human fetal tissue, human pluripotent-stem-cell-derived cells, and patient myocardium represent different levels of validation rather than interchangeable “human evidence” [1,25,26,32,33].

**Figure 1:**
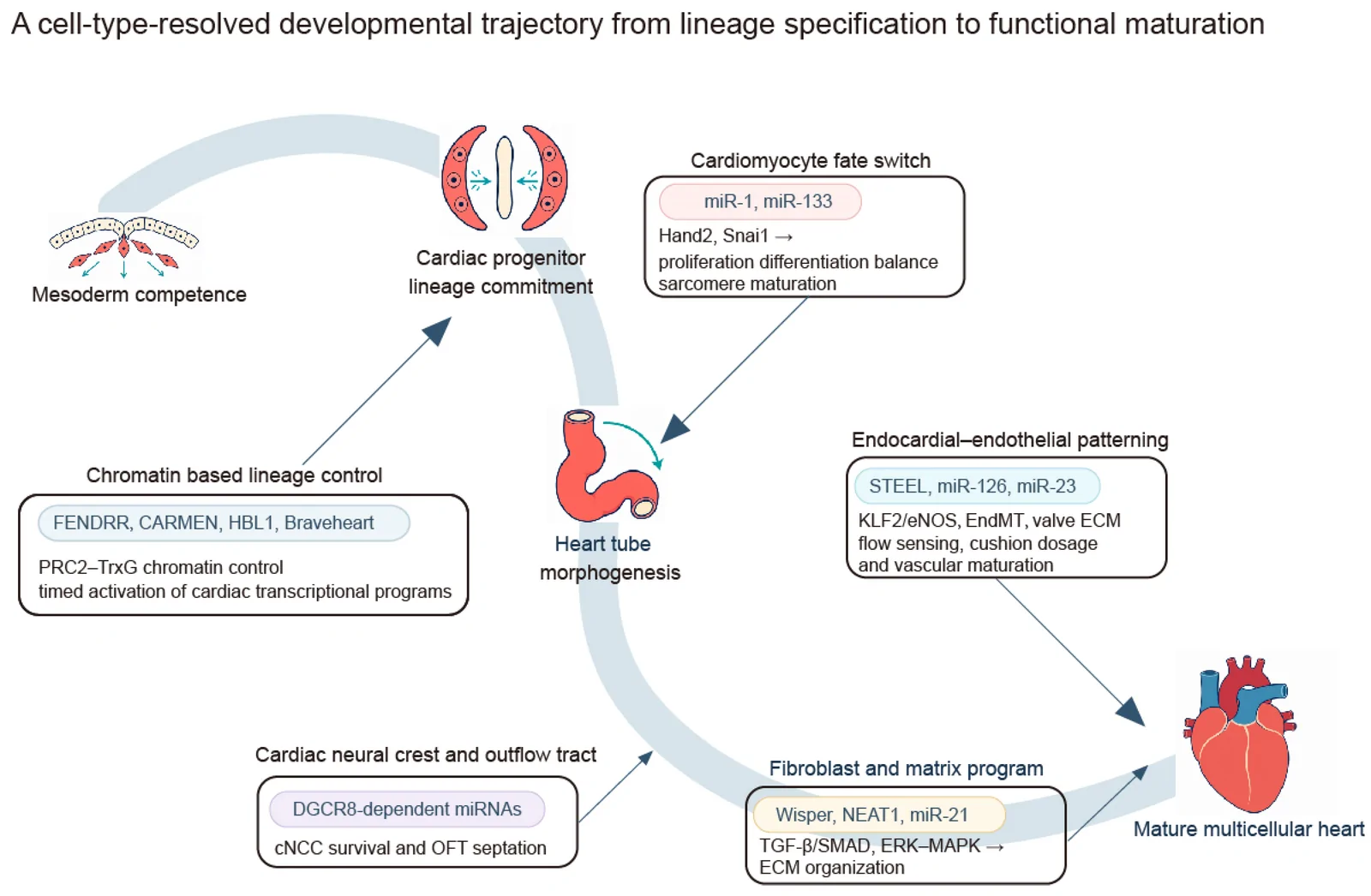
Cell-type-resolved ncRNA regulation during cardiac development. ncRNAs coordinate lineage specification, cardiomyocyte maturation, endothelial patterning, extracellular-matrix organization, and outflow-tract development from mesoderm to the mature heart. The broad pale arrow indicates developmental progression, whereas thin arrows link each ncRNA module to the affected transition. Abbreviations: cNCC, cardiac neural crest cell; ECM, extracellular matrix; ERK–MAPK, extracellular signal-regulated kinase–mitogen-activated protein kinase; OFT, outflow tract; PRC2, Polycomb repressive complex 2; TGF-β, transforming growth factor beta; TrxG, Trithorax group.

Four model classes are distinguished. Lineage-restricted perturbation in an intact embryo can link an RNA pathway to morphogenesis, although pleiotropy and incomplete lineage specificity remain possible. Primary fetal or neonatal cardiomyocytes test differentiation and early maturation outside full organ architecture. Directed ESC/iPSC differentiation recapitulates selected specification steps in vitro but does not reproduce embryonic hemodynamics, multicellular anatomy, placental influences, or the complete developmental timetable. Adult pressure-overload, infarction, and reprogramming models test stress responses or induced cell-state conversion, not normal embryogenesis. Conclusions below are limited to the model class in which they were obtained. The developmental trajectory and principal cell-type modules are summarized in Figure 1.

### 2.1. Cardiac Progenitors—From Mesodermal Competence to Lineage Commitment

#### 2.1.1. Establishing Chromatin Competence Before Cardiac Lineage Commitment

Before cardiac genes can be activated, mesodermal progenitors must establish a chromatin state that permits lineage commitment without premature differentiation. Polycomb repressive complex 2 (PRC2) generally deposits the repressive H3K27me3 mark, whereas Trithorax-group/mixed-lineage leukemia (MLL) complexes promote the activating H3K4me3 mark. FENDRR is included because it connects this opposing chromatin machinery to an intact developmental phenotype in mouse lateral-plate mesoderm. *Fendrr* loss altered PRC2 occupancy and H3K27me3/H3K4me3 at selected cardiac regulatory loci and produced cardiac and body-wall defects with altered *Gata6* and *Nkx2-5* expression. The data support a role in coordinating chromatin state with early mesodermal differentiation; they do not prove a graded epigenetic dosage mechanism because transcript-dose series, locus-specific rescue, and temporally resolved chromatin-to-phenotype experiments were not performed [7].

CARMEN addresses a related developmental question in a different model: whether a human fetal cardiac-progenitor locus is required for specification in culture. Knockdown impaired cardiac specification and differentiation, and the locus was reported to interact with PRC2 components. Isoform-resolved work further showed that CARMEN-201 contains a MIRc element that recruits REST and shifts the balance between cardiomyocyte and smooth-muscle programs. CARMEN is therefore not a human equivalent of FENDRR or part of one conserved FENDRR-CARMEN pathway. It is a separate, isoform-dependent example from human progenitor cells, and its intact-embryo function remains untested [8,25].

HBL1 tests an earlier in vitro state: chromatin restraint in undifferentiated human pluripotent stem cells before and during directed cardiogenesis. JARID2 is a PRC2-associated targeting and regulatory factor, whereas EED is a core PRC2 subunit required for propagation of H3K27 methylation. HBL1 interacted with JARID2 and EED; its depletion reduced PRC2 occupancy at cardiogenic loci and accelerated cardiomyocyte differentiation. This establishes a mechanism within the human pluripotent stem cell (hPSC) differentiation system, not in an intact human embryo. FENDRR, CARMEN, and HBL1 are thus retained as three model-resolved examples of how lncRNAs can influence chromatin competence, rather than as members of one universal developmental pathway [26].

#### 2.1.2. Balancing Progenitor Expansion and Differentiation in the First and Second Heart Fields

The first heart field contributes predominantly to the left ventricle, whereas the second heart field adds progenitors to the right ventricle and outflow tract. Their expansion must be sufficient before differentiation; premature withdrawal reduces chamber growth, whereas delayed differentiation disrupts structural assembly. In mouse embryos, miR-1 helps set this balance by repressing *Hand2*, a transcription factor required for right-ventricular progenitor expansion. Cardiac miR-1 overexpression caused premature differentiation and thin ventricular walls, whereas miR-1-2 loss increased *Hand2* expression, cardiomyocyte proliferation, and septal defects. Compound miR-1-1/miR-1-2 loss caused embryonic lethality and sarcomere disruption, supporting a dose-sensitive developmental requirement rather than a simple on/off switch [10,11].

miR-133a answers a complementary embryonic question. Double deletion of miR-133a-1 and miR-133a-2 caused embryonic or neonatal lethality, ventricular septal defects, right-ventricular dilation, increased apoptosis, and inappropriate smooth-muscle-gene expression, indicating that progenitor survival and lineage restriction must accompany miR-1-driven differentiation. By contrast, addition of miR-133a to a GMT fibroblast-reprogramming cocktail increased production of induced cardiomyocyte-like cells through *Snai1* repression. That result demonstrates induced cell-state conversion in cultured mouse fibroblasts; it is not evidence for first- or second-heart-field development and is not used to infer embryonic morphogenesis [12,27].

#### 2.1.3. Directed hPSC Differentiation and circRNA Candidates: Association Versus Developmental Function

Directed hPSC differentiation provides a controlled sequence from pluripotency through mesoderm, cardiac progenitors, and cardiomyocyte-like cells. Stage-resolved RNA sequencing showed changing circRNA profiles across this in vitro sequence, identifying candidate markers of specification but not proving a role in embryonic heart morphogenesis. circNCX1 was examined because it is cardiac enriched and can restrict cardiomyocyte cell-cycle activity: forced expression reduced cell-cycle readouts through a proposed BRG1-FBXW7 protein-bridging mechanism and lower BMP10. This finding informs proliferative exit in experimental cardiomyocytes. It does not establish circNCX1 as a regulator of heart-field specification, heart-tube formation, or chamber morphogenesis, and its lower abundance in zebrafish is a cross-species association rather than an explanation of zebrafish regeneration [1].

The evidentiary boundary is important because circularity alone does not establish function. A sponge mechanism requires endogenous copies per cell, accessible binding-site number, cytoplasmic colocalization, AGO occupancy, loss-of-function, and rescue at physiological abundance. A protein-bridging mechanism requires endogenous localization, complex occupancy, disruption of the relevant interfaces, and rescue. circNCX1 satisfies several functional criteria in experimental cardiomyocytes, but its role remains an in vitro or injury-related cardiac-cell-cycle mechanism rather than demonstrated embryonic morphogenesis [1].

### 2.2. Cardiomyocyte Differentiation and Maturation: Separating Early Differentiation, Postnatal Maturation, and Adult Stress

#### 2.2.1. Coupling Contractile-Gene Programs to Electrical Conduction: miR-208a

Electrical maturation requires impulse propagation to remain coordinated with the contractile program. Connexin 40, encoded by *GJA5*, forms gap junctions in atrial and specialized conduction tissues; HOPX/Hop is a cardiac transcriptional coregulator linked to conduction-gene expression; and the electrocardiographic PR interval reports atrioventricular conduction time. miR-208a is relevant because it is encoded within *Myh6* and therefore links a contractile host gene to this electrical program. In transgenic and knockout mice, altered miR-208a changed hypertrophic growth, Hop and Cx40 expression, PR interval, and arrhythmia susceptibility. These are mouse cardiac-conduction and stress phenotypes, not evidence that miR-208a alone controls all aspects of human cardiomyocyte maturation [28].

The study proposed a GATA4-Hop-Cx40 cascade and showed that miR-208-family expression parallels the thyroid-hormone-dependent switch of myosin host genes. This provides a mechanistic bridge between contractile-gene regulation and conduction. However, the complete cascade was not resolved by lineage- and time-specific rescue at every step, and Cx40 is not a general marker of working-ventricular cardiomyocyte maturity. The evidence therefore supports a model of myosin-coupled electrical regulation in mice rather than a universal fetal-to-adult maturation switch [28].

#### 2.2.2. Braveheart and MIAT Inform Different Processes and Experimental Models

Braveheart is relevant to early lineage commitment, not principally to terminal maturation. In mouse embryonic-stem-cell differentiation, its depletion impaired activation of the cardiovascular gene network upstream of *Mesp1*, and interaction with PRC2 was reported. Reduced sarcomeric-gene expression after depletion in neonatal cardiomyocytes suggests a possible post-commitment effect, but poor conservation and the absence of long-term lineage-maintenance experiments limit that interpretation. The strongest conclusion is therefore that Braveheart facilitates early mouse cardiovascular differentiation in vitro [9].

MIAT informs a different process. Although its abundance changes during hPSC differentiation, the cited causal experiment used adult mice under pressure overload: *Miat* deletion attenuated pathological hypertrophy and altered calcium-handling and splicing-associated readouts. Adult stress remodeling is neither embryonic differentiation nor normal postnatal maturation. MIAT is retained here only to make that boundary explicit; its adult phenotype does not establish a developmental maturation mechanism and the two lncRNAs should not be interpreted as discordant regulators of the same process [34].

#### 2.2.3. The piRNA/PIWI Evidence Boundary: Cardiac Expression Does Not Establish Terminal Differentiation

The relevant question is whether the presence of PIWI proteins or piRNA-sized reads in cardiac samples demonstrates a role in cardiomyocyte differentiation. It does not. PIWI-piRNA systems are established mediators of germline transposon control, and cardiac expression surveys plus changes in piR-1078 during differentiation are descriptive. PIWIL2-dependent transposon silencing in iPSCs occurs during an early pluripotent-to-mesoderm transition and cannot be extrapolated to terminal cardiomyocyte differentiation without lineage-restricted loss-of-function, target identification, and rescue [35].

HAAPIR provides causal cardiac evidence in another setting: adult myocardial infarction and cardiomyocyte death through NAT10-mediated ac4C modification of *Tfec* mRNA. That study supports a piRNA-linked injury mechanism, not embryonic development or terminal maturation. We therefore treat piRNA/PIWI signaling as an evidence gap in developmental cardiology rather than as an established layer of cardiomyocyte maturation [36].

### 2.3. Endocardial and Endothelial Cells—Shaping Chamber Topology via Flow-Responsive ncRNAs

#### 2.3.1. Shear-Stress-Induced lncRNA STEEL and Valve Morphogenesis

Endocardial and endothelial cells experience flow and growth-factor cues that influence chamber, valve, and vascular morphogenesis. STEEL provides evidence that an endothelial-enriched lncRNA can regulate eNOS- and KLF2-associated transcription in cultured endothelial cells and that its abundance changes under laminar flow. These findings support a flow-responsive endothelial circuit, but the experiments did not directly test embryonic valve morphogenesis or prove that STEEL mediates a binary switch between endothelial states in vivo [13].

The developmental relevance of a flow-responsive lncRNA must therefore be established at the level of the structure being formed. Endothelial target engagement in culture is mechanistic evidence for vascular-state regulation; lineage-restricted perturbation during cushion or vessel formation, followed by morphological and hemodynamic rescue, would be required to assign a direct morphogenetic function [13].

#### 2.3.2. Endothelial-to-Mesenchymal Transition (EndMT) and Cushion Matrix: Distinguishing Pathway Plausibility from In Vivo Morphogenesis

Valve and septal morphogenesis requires a subset of endocardial cells to undergo endothelial-to-mesenchymal transition, invade the cardiac cushions, and remodel a hyaluronan-rich extracellular matrix. miR-126 is included because it inhibited EndMT through PIK3R2-PI3K/AKT signaling in endothelial-progenitor-cell culture, providing pathway plausibility for control of the endothelial state. The experiment did not test an embryonic cushion. By contrast, miR-23 perturbation in zebrafish altered *Has2*-dependent cushion matrix and produced valve malformation, directly linking an ncRNA perturbation to a morphogenetic endpoint. These studies therefore occupy different evidence levels and do not establish a common miRNA pathway [29,30].

### 2.4. Developmental Fibroblast and Matrix Programs: A Defined Evidence Gap

Cardiac fibroblasts arise mainly from epicardial and endocardial lineages and build matrices required for myocardial compaction, valve remodeling, and coronary support. However, the available ncRNA studies cited in this review predominantly examine activated adult fibroblasts after infarction, pressure overload, aortic stenosis, or dilated cardiomyopathy. Wisper, NEAT1, and miR-21 are therefore discussed as adult fibrotic mechanisms in Section 4.3.4, not as evidence for embryonic fibroblast specification. Direct developmental claims will require lineage-resolved perturbation during epicardial or endocardial fibroblast emergence, followed by matrix, coronary, and chamber-level phenotyping. The absence of such evidence is itself the conclusion of this subsection [14].

### 2.5. miRNA Biogenesis in Cardiac Neural Crest and Outflow-Tract Morphogenesis

Cardiac neural crest cells arise from the dorsal neural tube, migrate through the pharyngeal arches, and contribute to aortic-arch remodeling, great-vessel smooth muscle, and septation of the cardiac outflow tract. Their developmental trajectory is distinct from both cardiac mesoderm and the myocardial conduction program. ncRNA evidence in this lineage remains limited, so it is important to distinguish dependence on global miRNA processing from the function of an individual mature miRNA [32].

DGCR8 is the double-stranded-RNA-binding component of the nuclear DROSHA-DGCR8 Microprocessor that converts primary miRNAs into precursor miRNAs. Wnt1-Cre marks premigratory and migrating neural crest and its descendants, including the cardiac neural crest cells that populate pharyngeal arches, great-vessel smooth muscle, and the outflow-tract septum; it also labels noncardiac neural-crest derivatives. Conditional *Dgcr8* loss in this lineage increased apoptosis in the caudal pharyngeal arches before outflow-tract entry and produced persistent truncus arteriosus, ventricular septal defects, and interrupted aortic arch. The experiment therefore shows that canonical miRNA biogenesis is required for survival of the Wnt1 lineage contributing to cardiovascular morphogenesis, but it does not identify the responsible mature miRNA and cannot assign every phenotype exclusively to cardiac neural crest [32].

Mef2c-AHF-Cre targets anterior second-heart-field mesoderm, which contributes myocardium and smooth muscle to the right ventricle and outflow tract and interacts with cardiac neural crest during septation. *Dgcr8* loss in this distinct lineage also caused outflow-tract and right-ventricular defects with reduced mature miRNAs. Together, the two models show that miRNA processing is required in interacting neural-crest and second-heart-field populations; they do not demonstrate a shared mature-miRNA mechanism. Because DGCR8 lies within the 22q11.2 deletion, a contribution to conotruncal disease is plausible, but independence from TBX1 dosage and the relevant human gene–dose relationship remain unresolved [33]. The model-resolved evidence discussed in this section is summarized in Table 1.

## 3. ncRNAs in Cardiac Regeneration: Reactivating Developmental Plasticity

The adult mammalian heart is not completely post-mitotic, but its endogenous cardiomyocyte renewal rate is too low to replace the large cell loss caused by myocardial infarction (MI). Retrospective radiocarbon dating indicates that human cardiomyocyte turnover declines from approximately 1% per year in young adults to less than 0.5% in old age, while lineage and isotope-labeling studies identify pre-existing cardiomyocytes as the principal source of new myocytes in the adult mammalian heart [16,17]. Consequently, ischemic injury is repaired predominantly by scar formation rather than restoration of contractile myocardium.

**Figure 2:**
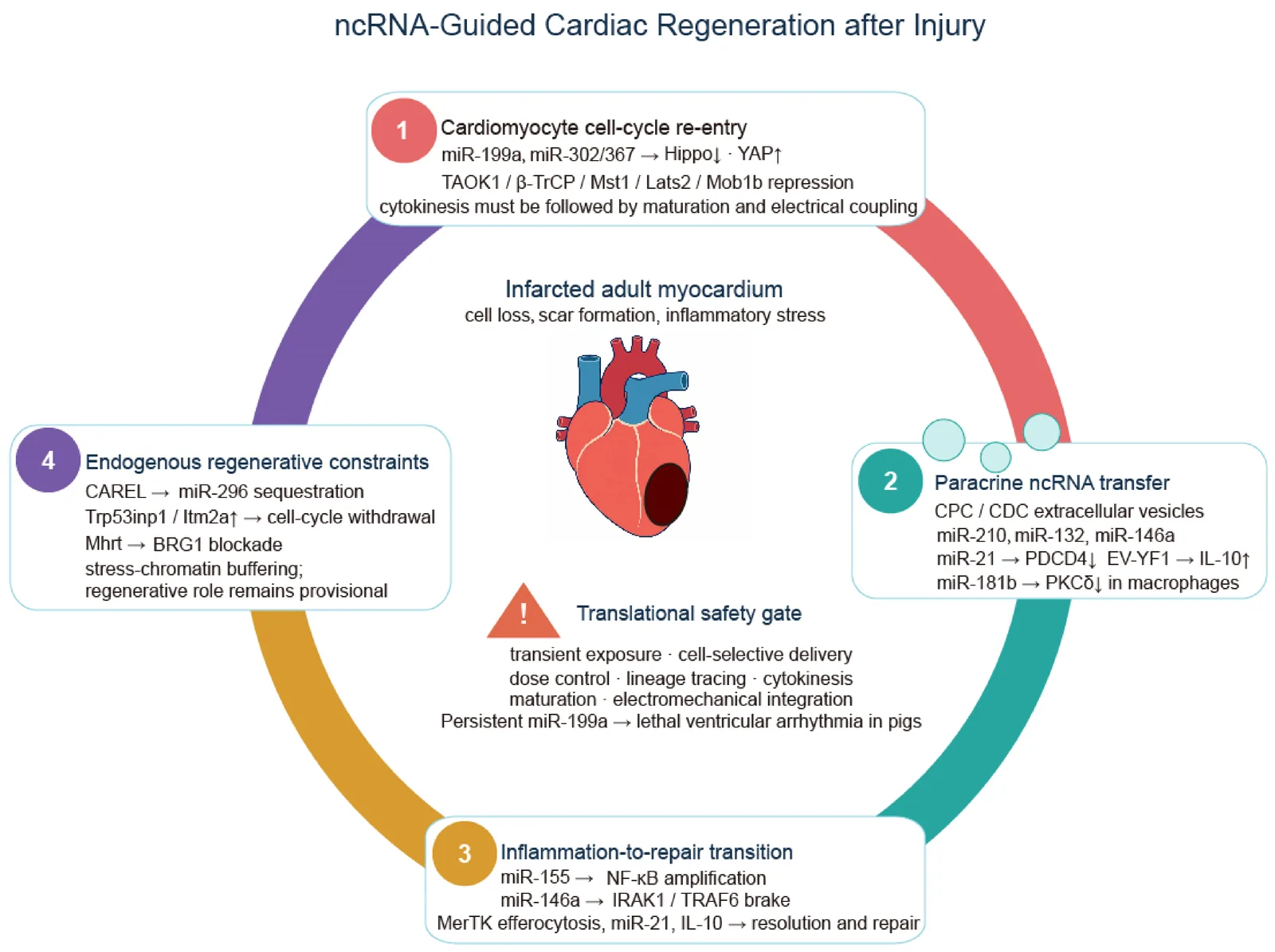
ncRNA-guided cardiac regeneration after injury. Cardiomyocyte cell-cycle re-entry, extracellular-vesicle signaling, immune resolution, and endogenous regenerative restraints jointly determine repair, while safe translation requires controlled delivery and functional maturation. The four numbered colored sectors represent cardiomyocyte cell-cycle re-entry, paracrine ncRNA transfer, the inflammation-to-repair transition, and endogenous regenerative constraints. The central heart depicts infarcted myocardium, and the warning triangle marks the translational safety gate. Abbreviations: CDC, cardiosphere-derived cell; CM, cardiomyocyte; CPC, cardiac progenitor cell; EV, extracellular vesicle.

This limitation is developmentally acquired. The neonatal mouse heart can regenerate after apical resection or MI during a short postnatal window, but this capacity is rapidly lost as cardiomyocytes undergo cell-cycle withdrawal, binucleation or polyploidization, metabolic maturation, and structural specialization [18]. ncRNAs are positioned at several control points in this transition: they tune Hippo–YAP activity within cardiomyocytes, mediate paracrine signaling through extracellular vesicles (EVs), and shape the inflammatory-to-reparative transition after injury. These mechanisms offer routes to reactivate repair, but they also reveal a central therapeutic constraint: proliferative or immune programs must be activated transiently and in the appropriate cell type (Figure 2).

For this review, cell-cycle re-entry denotes renewed expression or activity of cell-cycle machinery; DNA synthesis denotes S phase and may instead produce polyploidization, multinucleation, or DNA repair; mitosis denotes nuclear division; and cytokinesis denotes physical separation into daughter cells. Cardiomyocyte proliferation is used only when one cardiomyocyte generates two daughter cardiomyocytes and a net increase in cardiomyocyte number is supported. Cardioprotection refers to reduced cell death or infarct size without requiring new cardiomyocytes. True myocardial regeneration is the higher-order tissue outcome: lineage-restricted production of new cardiomyocytes must be coupled to sarcomere maturation, vascular support, electrical and mechanical integration, replacement rather than thinning of scar, durable functional recovery, and absence of arrhythmia [37]. No single marker, including Ki67, EdU, phospho-histone H3, or Aurora-B localization, establishes this full sequence.

Accordingly, evidence is interpreted by model and endpoint. Neonatal resection or infarction tests a transient mammalian regenerative state; adult rodent infarction tests repair in a scar-forming heart; large-animal infarction tests delivery, rhythm, and organ-scale safety; isolated neonatal cardiomyocytes and H9C2 cells test cell-cycle or survival pathways but cannot demonstrate remuscularization. Improved ejection fraction, smaller infarct size, angiogenesis, or reduced apoptosis may reflect cardioprotection or remodeling unless new cardiomyocyte production and integration are demonstrated independently.

### 3.1. Cardiomyocyte Cell-Cycle Re-Entry: ncRNA Control of Hippo–YAP Signaling

Hippo signaling is a major checkpoint of cardiomyocyte proliferation. In its active state, the MST1/2–SAV1–LATS1/2 kinase cascade phosphorylates YAP/TAZ, restricting their nuclear accumulation and TEAD-dependent transcription. Cardiac Hippo deficiency or expression of activated YAP increases adult cardiomyocyte cell-cycle activity and improves repair after experimental injury, whereas YAP loss impairs neonatal regeneration [38,39]. Pro-proliferative miRNAs exploit this pathway at multiple levels, thereby converting modest repression of several targets into a coordinated increase in nuclear YAP activity.

#### 3.1.1. miR-199a and the miR-302/367 Cluster: Experimental Inducers of Cardiomyocyte Proliferation

A high-content functional screen first identified miR-199a-3p and miR-590-3p as potent inducers of rodent cardiomyocyte DNA synthesis, mitosis, and cytokinesis; local delivery after MI increased cell-cycle markers and improved ventricular function in mice [19]. Mechanistic analysis subsequently showed that several pro-proliferative miRNAs converge on YAP. miR-199a-3p directly represses TAOK1 and β-TrCP, reducing Hippo-kinase activation and YAP degradation, and also targets Cofilin2 to favor an actin state permissive for YAP nuclear localization [40].

The same potency creates a safety problem. AAV6-mediated miR-199a expression in infarcted pig hearts initially reduced scar size and improved contractility, but persistent expression caused fatal ventricular arrhythmias in most treated animals [21]. This large-animal result demonstrates that evidence of cardiomyocyte cell-cycle activity is not sufficient to establish therapeutic regeneration: newly generated or dedifferentiated cells must also mature, couple electrically, and cease proliferating at the appropriate time.

The miR-302/367 cluster acts more directly on the Hippo kinase module. In mice, it represses Mst1, Lats2, and Mob1b, thereby increasing nuclear YAP and cardiomyocyte proliferation. Developmental deletion reduced cardiomyocyte proliferation, whereas postnatal re-expression or short-term administration of miRNA mimics reduced fibrosis and improved function after MI [20]. Sustained transgenic expression, however, produced cardiomyocyte dedifferentiation and ventricular dysfunction. The miR-199a and miR-302/367 studies therefore favor transient, dose-controlled RNA delivery over constitutive viral expression.

Accordingly, the evidence from these miRNA studies should be graded by endpoint. The rodent miR-199a/miR-590 screen supported DNA synthesis and late cell-cycle events, but it did not provide clonal lineage tracing, a direct count of new mononuclear daughter cardiomyocytes, or long-term electrical integration. The miR-302/367 experiments combined genetic and transient-mimic approaches with reduced fibrosis and improved ventricular function, yet functional improvement can arise from cytoprotection, altered remodeling, or angiogenesis as well as remuscularization. The porcine miR-199a study strengthened species translation and demonstrated early scar reduction and functional benefit, but the subsequent lethal arrhythmias show that neither cycling markers nor early ejection-fraction improvement prove safe, mature myocardial regeneration [19,20,21,40].

#### 3.1.2. lncRNA Control of Regenerative Competence: Direct Repair Evidence and Mechanistic Boundaries

CAREL was tested with *Myh6*-driven cardiomyocyte overexpression and adenoviral cardiac knockdown in neonatal apical-resection and neonatal or adult infarction models. The study measured mitotic and cytokinesis-associated cardiomyocyte markers together with scar and ventricular function, and miR-296 rescue supported the proposed Trp53inp1/Itm2a pathway. These data support a cardiomyocyte-directed effect on late cell-cycle activity and injury repair. They do not, however, provide clonal lineage tracing, a direct net increase in cardiomyocyte number, or long-term electrical-integration data. The competing-endogenous-RNA interpretation also remains less secure than the in vivo phenotype because absolute CAREL and miR-296 copies, accessible binding sites, and endogenous AGO occupancy were not quantified as a complete stoichiometric chain [41].

Mhrt belongs in this subsection only as a boundary case. In adult mice subjected to pressure overload, Mhrt bound the helicase domain of BRG1 and limited stress-induced chromatin remodeling, hypertrophy, and heart failure. This is strong evidence for chromatin-level cardioprotection during adult remodeling, but the experiment did not test infarct remuscularization, cardiomyocyte cytokinesis, or lineage-traced daughter-cell production. We therefore do not treat Mhrt as an endogenous brake on regeneration; instead, it illustrates that maintaining a permissive stress-chromatin state is biologically distinct from generating new myocardium [42].

LncBAR and CLIPPER provide newer, but still endpoint-limited, evidence. LncBAR gain- and loss-of-function experiments in neonatal and adult mouse injury models linked BRG1 stabilization to Ki67, phospho-histone H3, cytokinesis-associated readouts, scar reduction, and improved ventricular function; complementary human embryonic-stem-cell-derived cardiomyocyte experiments tested cell survival and cycling. CLIPPER was identified by a cardiomyocyte knockdown screen and connected in cis to LPIN1-dependent mitochondrial division, lower oxidative metabolism, reduced reactive oxygen species and DNA damage, cell-cycle activity, and functional recovery after mouse infarction. The authors describe these phenotypes as regeneration, but neither study established the complete sequence of lineage-traced daughter-cell formation, net cardiomyocyte gain, mature electromechanical integration, and long-term rhythm safety. We therefore refer to repair-associated cardiomyocyte cell-cycle activity unless the higher-order endpoints are shown [43,44].

The mechanistic certainty of these lncRNA studies is also unequal. CAREL has a cardiomyocyte-directed in vivo phenotype, but the proposed ceRNA explanation requires absolute CAREL and miR-296 abundance, subcellular colocalization, accessible binding-site number, AGO occupancy, and dose-dependent rescue at endogenous expression; these quantities were not established as a complete stoichiometric chain. Mhrt has strong chromatin-binding and cardioprotective evidence but no direct cytokinesis endpoint. LncBAR has gain- and loss-of-function support in neonatal and adult mouse injury models and complementary human stem-cell-derived cardiomyocyte data, but no human myocardial injury or electrical-integration validation. CLIPPER links a conserved locus to LPIN1-dependent mitochondrial remodeling in mouse and cultured human systems, yet clinical myocardial target engagement and durable safety remain unknown. Thus, the in vivo phenotypes are generally stronger than any claim that one proposed molecular interaction alone explains regeneration [41,42,43,44].

### 3.2. Paracrine ncRNA Signaling in the Cardiac Progenitor and Stem-Cell Niche

Cell populations historically described as cardiac progenitor cells (CPCs) or cardiosphere-derived cells (CDCs) show limited durable engraftment and little convincing direct cardiomyogenic replacement after transplantation. Their relevance here is therefore paracrine: conditioned small-EV preparations can change cardiomyocyte survival, endothelial behavior, and macrophage state. The direct endpoints in the cited studies are predominantly apoptosis, infarct size, angiogenesis, inflammatory signaling, or ventricular function. These outcomes are components of repair, but none by itself demonstrates that a progenitor-derived RNA cargo generated new, mature cardiomyocytes [22,23,24,45,46,47].

#### 3.2.1. Extracellular-Vesicle ncRNAs from Cardiac Progenitor and Cardiosphere-Derived Cells

Early studies showed that CPC-derived EVs are taken up by cardiomyoblasts and reduce apoptosis after oxidative stress or myocardial ischemia/reperfusion; miR-451 was enriched in the vesicle fraction [45]. Human CPC-EVs similarly reduced cardiomyocyte apoptosis, enhanced endothelial tube formation, and improved ventricular function after MI. Their enriched miRNA cargo included miR-210, which repressed ephrin-A3 and PTP1B in cardiomyocytic cells, and miR-132, which promoted endothelial responses through RasGAP-p120 repression [22].

CDC-derived exosomes reproduced several benefits of CDC transplantation in infarcted mouse hearts, whereas inhibition of exosome production reduced those benefits. miR-146a was enriched in CDC exosomes, and a miR-146a mimic reproduced part, but not all, of the exosomal phenotype [23]. Under oxidative stress, Sca1-positive CPCs released exosomes enriched in miR-21; transfer to cardiomyoblasts suppressed PDCD4 and reduced apoptosis [46]. Because the latter study relied mainly on H9C2 cells and oxidative-stress assays, it supports a survival mechanism rather than direct evidence of cardiomyocyte regeneration.

#### 3.2.2. Cargo Selection, Cellular Uptake, and Intercellular Reprogramming in the Cardiac Niche

EV-mediated signaling extends beyond miRNAs. A Y RNA fragment, EV-YF1, is abundant in CDC-EVs and is transferred to macrophages, where it increases IL-10 expression; EV-YF1 administration reduced infarct size after ischemia/reperfusion [47]. In rat and pig MI models, CDC exosomes also altered macrophage transcriptional state. Functional loading experiments identified miR-181b transfer and repression of PKCδ as a mechanism contributing to this cardioprotective macrophage phenotype [24]. Thus, EV cargo can reprogram the behavior of several recipient cell types and coordinate survival, vascular, and immune responses.

The term “intercellular reprogramming” should nevertheless be used narrowly. The cited studies show phenotypic modulation, not stable lineage conversion of recipient cells into cardiomyocytes. Interpretation is further limited by heterogeneous EV isolation methods, incomplete proof of endosomal origin, variable RNA copy number per vesicle, and strong dependence of cargo on donor-cell state. For clinical translation, defined RNA cargo, potency assays, biodistribution, and batch-controlled manufacturing will be more informative than vesicle number alone.

EV evidence in this literature spans several levels of rigor. The early mouse CPC study by Chen et al. confirmed a small-vesicle preparation by electron microscopy and CD63 and demonstrated uptake by H9C2 cells, but miR-451 was identified by enrichment rather than cargo-specific depletion/rescue, and neither recovery efficiency nor absolute copies delivered per recipient cell were measured [45]. Barile et al. used human atrial-appendage-derived CPCs, compared CPC-EVs with fibroblast EVs, depleted EVs from conditioned medium, and demonstrated repression of miR-210 and miR-132 targets in cardiomyocytic and endothelial cells; however, in vivo recipient-cell uptake and per-vesicle RNA stoichiometry were not established [22]. Ibrahim et al. linked CDC exosome production to benefit and showed that a miR-146a mimic reproduced only part of the phenotype, appropriately indicating multi-cargo activity rather than a single-miRNA mechanism [23]. Xiao et al. added nanoparticle tracking and a miR-21-PDCD4 axis, but relied largely on mouse CPCs and H9C2 cardiomyoblasts without primary adult cardiomyocyte, absolute-copy, or in vivo target-engagement evidence [46].

The EV-YF1 and miR-181b studies provide stronger recipient-cell evidence but still do not prove myocardial regeneration. Cambier et al. identified EV-YF1 as an abundant CDC-EV RNA, tracked transfer to macrophages, and linked it to IL-10 secretion and acute infarct limitation; this establishes macrophage-mediated cardioprotection, not new cardiomyocyte formation [47]. de Couto et al. combined rat and pig ischemia-reperfusion models, miR-181b loading, macrophage transcriptomics, PKC-delta suppression, and adoptive transfer, providing the strongest cargo-to-recipient causal chain among the cited studies [24]. Even here, absolute endogenous miR-181b copies per vesicle and per cardiac macrophage, first-pass tissue retention, and durability beyond acute cardioprotection were incompletely resolved. Across studies, compliance with MISEV2023 requires reporting source-cell conditioning, separation recovery and purity, orthogonal particle and protein characterization, non-vesicular contaminants, RNase/protease protection, dose normalized to producer-cell equivalents and particles, biodistribution, and recipient-cell target engagement [2]. This is important because bulk enrichment does not establish a functional dose: quantitative analyses in other systems found substantially fewer than one copy of even abundant miRNAs per exosome on average [3].

### 3.3. ncRNA Coordination of the Inflammation-to-Repair Transition

Macrophages are not simply debris-clearing cells. Their depletion prevents neonatal mouse heart regeneration and neoangiogenesis, demonstrating that an appropriate immune response is required for scar-free repair [48]. In adult MI, recruited inflammatory monocytes and resident or monocyte-derived reparative macrophages occupy overlapping, dynamically changing states; the binary M1/M2 nomenclature is therefore useful as shorthand but does not capture the full in vivo spectrum. ncRNAs influence this transition both cell-autonomously and through EV-mediated communication.

#### 3.3.1. Fibroblast-Macrophage lncRNA Signaling and Efferocytosis: Cell-Autonomy Boundaries

The lncRNA evidence does not support a single macrophage-autonomous pathway. Mirt1 was induced mainly in mouse cardiac fibroblasts; adenoviral knockdown altered fibroblast NF-κB signaling, reduced macrophage migration and inflammatory-cell infiltration, and improved infarct outcome. It therefore supports fibroblast-to-immune communication, not an endogenous macrophage lncRNA mechanism. MALAT1 was enriched in infarct-associated macrophages and was linked by pull-down and immunoprecipitation to an EZH2-H3K27me3-PPAR-γ axis, but the in vivo inhibition was not macrophage-restricted and whole-infarct changes could partly reflect altered cell composition. lincRNA-Cox2 has stronger chromatin-remodeling evidence in stimulated mouse macrophages, but outside a cardiac injury model. Thus, only MALAT1 currently provides cardiac macrophage-associated mechanistic evidence, and even that falls short of lineage-restricted in vivo causality [49,50,51].

Efferocytosis provides a biologically relevant bridge from inflammation to repair. MerTK loss in mouse myeloid cells impaired clearance of apoptotic cardiomyocytes, delayed inflammatory resolution, and worsened ventricular function after infarction, directly establishing the cardiac process. The miR-21 mechanism was demonstrated separately in human peripheral-blood-monocyte-derived macrophages exposed to apoptotic cells in a wound-inflammation model: miR-21 repressed PTEN and PDCD4, reduced NF-κB output, and favored IL-10. It is a plausible molecular explanation for a post-efferocytosis state, but it was not tested during cardiomyocyte efferocytosis in an infarcted heart. We therefore do not present miR-21 as a validated cardiac efferocytosis regulator [52,53].

#### 3.3.2. miR-155 and miR-146a: A Counter-Regulatory Pair Governing Innate Immune Resolution

The miR-155/miR-146a counter-regulatory logic was established mainly in activated human monocytes and genetic mouse macrophage systems outside myocardial infarction. In those systems, early miR-155 amplifies inflammatory output by repressing negative regulators such as SOCS1 or SHIP1, whereas induced miR-146a targets IRAK1 and TRAF6 and limits Toll-like-receptor signaling. These studies define a transferable innate-immune circuit, not a direct map of infarct macrophage states. Applying the circuit to the injured heart therefore requires time-resolved, macrophage-subset-specific perturbation rather than treating miR-155 and miR-146a as a simple M1/M2 switch [54,55].

Cardiac studies narrow, but do not eliminate, this uncertainty. Systemic miR-155 inhibition after mouse infarction reduced macrophage inflammatory signaling and cardiomyocyte endoplasmic-reticulum stress and apoptosis in a SOCS1-associated pathway; because the antagomir was not macrophage-specific, the in vivo result cannot assign cell autonomy. A later M1-macrophage-EV study showed vesicle uptake and transfer of miR-155 to cultured cardiomyocytes, with IL-6R/JAK2/STAT3 target engagement. Its in vitro and peri-infarct readouts were Ki67 and phospho-histone H3, which indicate cell-cycle entry or mitosis but not completed cytokinesis, ploidy-normal daughter cells, or myocardial regeneration. Native-EV miR-155 depletion and rescue at measured physiological copy number were also not shown. miR-146a, in turn, reproduced only part of the CDC-EV phenotype. These results support context-dependent immune-to-cardiomyocyte signaling, not a completed regenerative mechanism [23,56,57].

Immune modulation and EV-mediated cytoprotection should therefore be reported as components of cardiac repair unless cardiomyocyte production is demonstrated independently. Reduced inflammatory infiltration, enhanced efferocytosis, angiogenesis, smaller infarct size, or improved ejection fraction can improve healing without replacing scar with new myocardium. A regeneration claim should combine cardiomyocyte-restricted lineage or clonal tracing with direct cytokinesis and ploidy analysis, quantitative cardiomyocyte number, scar composition and thickness, capillary support, sarcomere and metabolic maturation, conduction and calcium-handling measurements, arrhythmia surveillance, and durable functional benefit [24,37,47,48,52]. The cell-, species-, and endpoint-resolved evidence is summarized in Table 2.

## 4. ncRNAs Across the Cardiovascular Continuum—Shared Regulatory Modules in Vascular and Cardiac Disease

This section asks how ncRNA-regulated cell states propagate from vessels to myocardium and from systemic load to cardiac outcome. Atherosclerosis is included because endothelial dysfunction, foam-cell biology, smooth-muscle remodeling, and plaque disruption determine coronary ischemic injury. Hypertension is included because vascular resistance and neurohumoral activation impose the load that drives myocardial hypertrophy and failure. These are not excursions from cardiac biology; they are upstream components of the vascular–cardiac disease continuum [58,59,60,61,62,63,64,65,66,67,68,69,70,71,72,73].

Disease selection followed three criteria: a direct effect on myocardial perfusion, load, rhythm, metabolism, or ventricular function; reuse of a regulatory operation introduced in development or repair; and mechanistic or human evidence in a defined cardiovascular compartment. Renal ncRNAs are discussed only where they regulate renin–angiotensin signaling or blood pressure and thereby alter cardiac afterload. Pulmonary arterial hypertension is included only as a model of pulmonary vascular remodeling coupled to right-ventricular adaptation. The review does not attempt to cover renal or pulmonary disease as independent organ systems [74,75,76,77,78].

**Figure 3:**
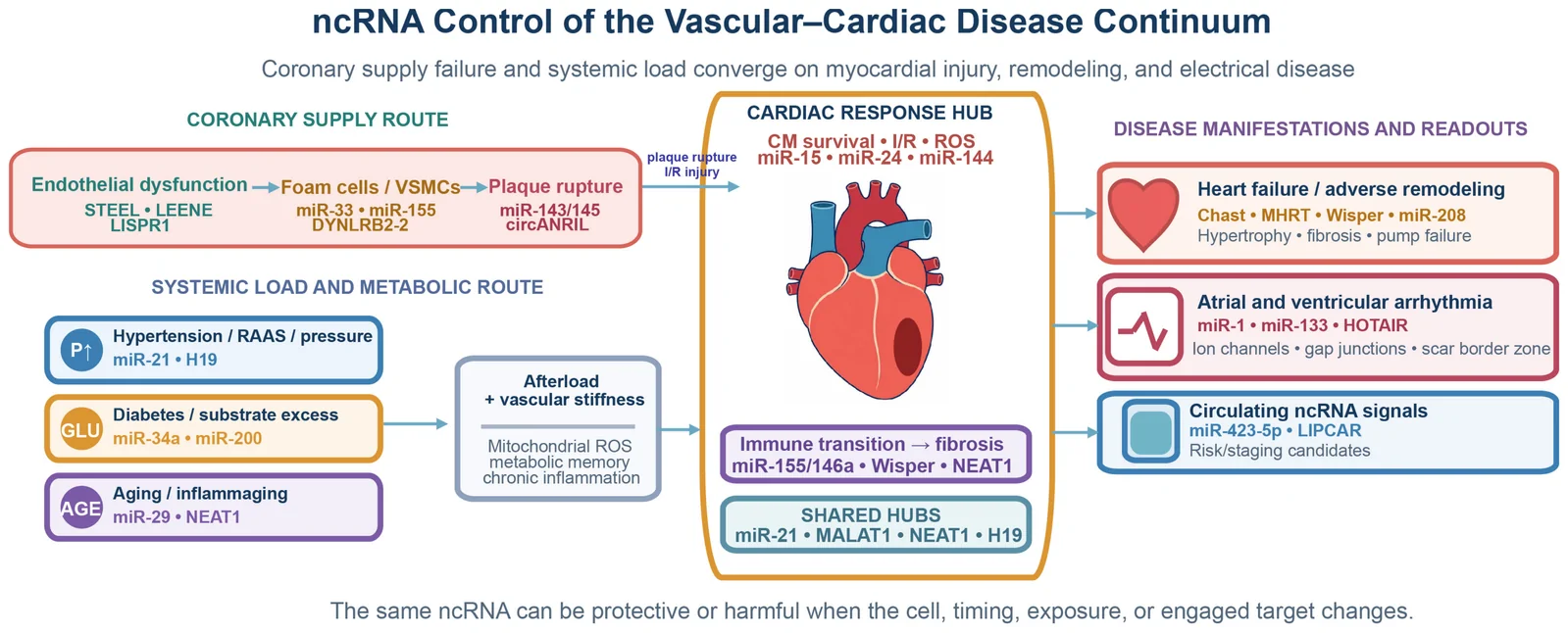
Dual-route map of ncRNA regulation across the vascular–cardiac disease continuum. Coronary supply failure and systemic pressure, metabolic, and aging-related stress converge on myocardial injury and a shared cardiac response hub, leading to heart failure, arrhythmia, and circulating ncRNA signals. Representative ncRNAs are shown within each module; their effects depend on cell identity, disease phase, dose, localization, and physiological target engagement. Arrows indicate predominant propagation of injury rather than exclusive causality; colors distinguish biological modules, the central box integrates convergent cardiac responses, and the right-hand boxes show clinical outcomes and readouts. Abbreviations: AGE, aging; CM, cardiomyocyte; GLU, glucose/substrate excess; I/R, ischemia/reperfusion; MI, myocardial infarction; P↑, pressure overload; RAAS, renin–angiotensin–aldosterone system; ROS, reactive oxygen species; VSMC, vascular smooth muscle cell.

Rather than treating each diagnosis as an isolated list of molecules, the subsections test six recurring modules: endothelial sensing and lipid handling; innate immune activation and resolution; cardiomyocyte survival and mitochondrial stress; fibroblast and smooth-muscle state transitions; electrical remodeling; and systemic pressure or metabolic load. Bulk-tissue expression remains hypothesis-generating because disease changes cell composition. Causal priority is therefore given to localization, cell-restricted perturbation, target rescue, appropriate species, and human target engagement [2,3,37]. A process-based map of these linked modules is provided in Figure 3.

Each disease subsection therefore begins with the failed cardiovascular process and then asks what an ncRNA experiment actually establishes in the sampled cell, species, and phase. A target name, whole-tissue expression change, or reporter interaction is not treated as self-explanatory evidence of disease causality. This same sequence—process, model, direct result, inference, and limitation—is applied to vascular, myocardial, immune, and circulating-RNA studies.

### 4.1. Atherosclerosis—ncRNAs at the Interface of Lipid Metabolism, Inflammation, and Plaque Biology

Atherosclerosis is initiated by disturbed endothelial homeostasis and sustained by lipid-loaded macrophages, smooth-muscle-cell plasticity, and maladaptive inflammation. ncRNAs participate at each level, but plaque-stage and cell-specific effects are essential: the same RNA may restrain early inflammation yet impair later resolution, or may show opposite associations in tissue and plasma.

#### 4.1.1. Endothelial Activation and Dysfunction: Shear-Stress-Responsive lncRNAs (STEEL, LEENE, and LISPR1)

Atheroprotective laminar flow maintains endothelial quiescence, barrier integrity, and nitric-oxide-dependent vasodilation. KLF2 is a flow-induced transcription factor that stabilizes this state; eNOS produces nitric oxide; and S1PR1 supports endothelial barrier signaling. STEEL is relevant because it regulates KLF2- and eNOS-associated transcription in endothelial cells, LEENE promotes eNOS transcription, and LISPR1 supports S1PR1 expression under laminar flow. These experiments establish endothelial target engagement in culture or vascular models. They do not yet show that manipulation of any one lncRNA prevents plaque events in humans [13,79,80].

#### 4.1.2. Macrophage Foam-Cell Formation: miR-33, miR-155, and lincRNA-DYNLRB2-2 in Cholesterol Efflux

A macrophage becomes a foam cell when modified-lipoprotein uptake exceeds cholesterol export, with ABCA1-mediated efflux providing a major protective route. miR-33 is relevant because it is encoded within SREBF transcripts and represses ABCA1 and other lipid-handling genes; inhibition increases efflux and can promote plaque regression in experimental models. miR-155 instead modifies inflammatory state, and its net effect changes with lesion phase. lincRNA-DYNLRB2-2 increased ABCA1-linked efflux by reducing TLR2 signaling in macrophage models. These are distinct lipid-handling and inflammatory mechanisms; the nonstandard label lncRNA-DYNAMO is not retained [58,59,60,61,62].

#### 4.1.3. Vascular Smooth-Muscle-Cell Phenotypic Switching: The miR-143/145 Axis

The miR-143/145 cluster promotes the contractile vascular smooth-muscle-cell (VSMC) phenotype by coordinating transcription factors and cytoskeletal targets. Genetic loss disrupts vessel-wall homeostasis and favors a less differentiated state, while gain-of-function experiments support restoration of contractile features [63,64]. Nevertheless, VSMC modulation cannot be reduced to blocking a contractile-to-synthetic transition: phenotypically modulated VSMCs can also generate fibrous-cap cells and stabilize plaques. Therapeutic interpretation must therefore distinguish lesion growth from cap integrity.

#### 4.1.4. Plaque Vulnerability and Rupture: circRNA and lncRNA Signals

Plaque transcriptomics has generated numerous ncRNA signatures, but most are cross-sectional and sensitive to cell composition. circANRIL provides a stronger mechanistic example: it binds PES1, impairs ribosomal RNA maturation, induces nucleolar stress, and limits proliferation in vascular cells, supporting an atheroprotective mechanism at the 9p21 locus [65]. This evidence does not justify using circANRIL alone to classify stable versus unstable plaques. Any vulnerability signature requires prospective validation against rupture-related clinical outcomes and adjustment for macrophage, VSMC, and endothelial abundance.

#### 4.1.5. Circulating ncRNAs as Liquid-Biopsy Markers of Subclinical Atherosclerosis

Circulating miRNA profiles differ between patients with coronary artery disease and controls, and early studies identified reproducible changes in endothelial-, platelet-, and inflammation-related miRNAs [66,67]. However, apparent signals are strongly affected by hemolysis, platelet activation, renal function, medication, extraction method, and normalization strategy. Current data support risk-association studies rather than screening of asymptomatic populations. Clinical development requires prespecified assays, external cohorts, comparison with established risk scores and imaging, and evidence of incremental decision value.

### 4.2. Ischemia–Reperfusion Injury—ncRNAs at the Life–Death Decision Point

Ischemia and reperfusion impose distinct stresses. Ischemia limits ATP production and ionic homeostasis, whereas reperfusion produces abrupt oxidative, calcium, mitochondrial, and inflammatory injury. ncRNA effects consequently depend on when and in which cell type an intervention is delivered; a target that limits acute cardiomyocyte death may have a different effect during later inflammation or scar formation.

#### 4.2.1. Phase-Specific ncRNA Dynamics: Ischemic Preconditioning Versus Lethal Reperfusion

Preconditioning induces a transient stress-adaptation program, whereas prolonged ischemia followed by reperfusion (I/R) activates cell-death and inflammatory networks. The miR-15 family increases after ischemic injury and represses prosurvival targets; systemic anti-miR treatment reduced infarct size and adverse remodeling in mice [68]. Conversely, miR-144 participates in remote preconditioning and is detectable in the circulation, illustrating that a protective RNA signal may be induced before lethal injury rather than after it [81]. These data argue for phase-resolved sampling and intervention rather than a single static I/R signature.

#### 4.2.2. MALAT1 and MIAT in Preclinical Cell-Death Models; HRCR as a Non-I/R Comparator

MALAT1 and MIAT were tested in preclinical injury systems, but neither study established a cell-specific mitochondrial-protection mechanism. In rats, whole-myocardium MALAT1 increased after ischemia-reperfusion and cardiac delivery of siRNA reduced infarct size, apoptosis, and dysfunction while restoring AKT phosphorylation; the study did not identify the expressing cell, demonstrate direct MALAT1-AKT binding, or test whether AKT activation was required by inhibition or rescue. The MIAT study combined H9c2 hypoxia-reoxygenation with lentiviral knockdown in mouse hearts and linked reduced apoptosis to lower NF-κB/PUMA signaling, again without lineage-restricted in vivo perturbation. HRCR was validated in pressure-overload hypertrophy through miR-223 sequestration, not in ischemia-reperfusion or mitochondrial ROS buffering. These three RNAs therefore cannot be presented as one established mitochondrial-protection axis [82,83,84].

#### 4.2.3. miR-21, miR-24, and miR-144: Cardioprotection with Context-Dependent Effects

The apparent direction of miR-21 depends on model, compartment, and timing. A rat-heart and H9c2 study associated miR-21 replacement with less apoptosis and PTEN-AKT signaling, but it did not establish cardiomyocyte-restricted in vivo target engagement. In contrast, the porcine study delivered intracoronary anti-miR-21 on days 5 and 19 after ischemia-reperfusion and evaluated the hearts at day 33; it reduced fibrosis, hypertrophy, macrophage and fibroblast abundance, and chronic dysfunction. It therefore addresses post-injury remodeling rather than acute reperfusion survival. miR-24 replacement after mouse infarction repressed BIM and reduced cardiomyocyte apoptosis, whereas the miR-144/451 cluster was required for preconditioning-associated protection in mice. These studies support cardioprotection or remodeling, not production of new myocardium [85,86,87,88].

#### 4.2.4. Vesicle-Dependent Transferable Cardioprotection Versus a Proven Inter-Organ RNA Cargo

The cited vesicle experiment used conditioned coronary effluent from isolated preconditioned donor rat hearts and transferred it to isolated recipient hearts. Removing the vesicle fraction abolished infarct-size protection, which supports a vesicle-dependent transferable factor but is not, by itself, an inter-organ experiment and did not identify an RNA cargo. The miR-144 study independently showed systemic cardioprotection and increased circulating miR-144 after limb preconditioning; importantly, most of the increase was associated with exosome-poor, AGO2-containing serum rather than a demonstrated vesicular delivery route. A specific EV-RNA mechanism would require cargo depletion from otherwise intact vesicles, physiological reconstitution, recipient-cell delivery, and target repression [81,89].

#### 4.2.5. Therapeutic Windows: Antisense Oligonucleotide (ASO) and miRNA-Mimic Delivery in Preclinical I/R Models

Anti-miR-15 treatment, miR-24 replacement, and modulation of miR-21 illustrate the main delivery strategies and their limitations [68,86,87]. Acute I/R favors rapidly available, reversible formulations, whereas persistent viral expression may extend beyond the protective window. Translational studies must define administration relative to reperfusion, biodistribution to cardiomyocytes versus fibroblasts and immune cells, renal and hepatic exposure, electrophysiological safety, and benefit on top of contemporary reperfusion therapy.

### 4.3. Heart Failure and Cardiac Hypertrophy—Rewriting the Fetal Gene Program

Pressure or volume overload reactivates developmental transcription, shifts myosin isoforms, increases extracellular-matrix deposition, and changes intercellular signaling. ncRNAs regulate each process, but mechanistic tissue studies and circulating biomarker studies answer different questions and should not be combined into a single causal narrative.

#### 4.3.1. Chast, MHRT, and LIPCAR in Hypertrophic and Dilated Remodeling

Chast, MHRT, and LIPCAR are grouped by clinical phenotype, not by one molecular mechanism. Chast addresses autophagy-associated hypertrophic remodeling: it is induced by pressure overload, represses PLEKHM1, and responds to antisense inhibition in mice. MHRT addresses chromatin control of fetal-gene reactivation by binding BRG1 and limiting pathological remodeling. LIPCAR addresses circulating prognosis and was associated with ventricular remodeling and cardiovascular mortality in patients. Only the first two provide tissue-level mechanistic perturbation; LIPCAR is a biomarker association. They should not be described collectively as a ceRNA network [42,69,70].

#### 4.3.2. miR-208a/b and miR-499: ncRNA Control of Myosin-Isoform and Stress Programs

The myosin-embedded myomiRs coordinate contractile-gene expression with cardiac stress responses. miR-208a, encoded by MYH6, is required for stress-dependent hypertrophy and β-myosin heavy-chain induction in mice [28]. miR-208b and miR-499 are encoded within MYH7 and MYH7B and reinforce slow-muscle and β-myosin programs through shared targets [71]. This intronic architecture creates a feed-forward system, but chronic inhibition may also disturb physiological conduction and contractile adaptation.

#### 4.3.3. circRNA Mechanisms in Dilated Cardiomyopathy: Sponging Versus Protein Scaffolding

Cardiac circRNAs can act through miRNA binding, RNA-binding proteins, transcriptional regulation, or translation, but disease-specific evidence is uneven. HRCR is a validated miR-223 sponge in hypertrophic remodeling [84]; it is not direct evidence for a protein-scaffolding mechanism in dilated cardiomyopathy (DCM). Multicenter profiling has identified circulating circRNA candidates that may help distinguish DCM etiologies [72], but most mechanistic scaffold claims remain preclinical and require stoichiometric target engagement and independent replication.

#### 4.3.4. Fibrotic Remodeling: Wisper, NEAT1, and Fibroblast–Cardiomyocyte Crosstalk

Wisper is enriched in cardiac fibroblasts and regulates fibrotic gene programs; antisense inhibition reduced fibrosis and preserved function in mouse cardiac injury models [14]. NEAT1 can promote fibroblast activation by recruiting EZH2 to repress SMAD7, thereby sustaining TGF-β/SMAD signaling [15]. Fibroblast-directed ncRNA therapy is therefore plausible, although cell-selective delivery remains essential because NEAT1 and other nuclear lncRNAs have distinct functions in endothelial, immune, and cardiomyocyte compartments.

#### 4.3.5. Plasma ncRNA Panels as Heart-Failure Staging Biomarkers

Circulating miR-423-5p and LIPCAR are among the most frequently replicated heart-failure candidates [70,73]. Their reported associations with diagnosis, remodeling, or mortality are encouraging but do not establish a staging test. Natriuretic peptides, renal function, rhythm, treatment, blood-cell composition, and pre-analytical handling all influence performance. A clinically useful panel must add discrimination or reclassification beyond established biomarkers and demonstrate transportability across heart-failure phenotypes.

### 4.4. Cardiac Arrhythmia—ncRNAs Reshaping the Electrophysiological Landscape

Arrhythmogenesis reflects the interaction of ion-channel expression, calcium handling, conduction, fibrosis, autonomic tone, and structural disease. ncRNAs can influence several components simultaneously, which makes them attractive mechanistic candidates but also creates proarrhythmic risk when expression is manipulated systemically.

#### 4.4.1. Ion-Channel Regulation: miR-1 and miR-133 in Kir2.1, Cx43, and HCN4 Expression

Electrical stability depends on Kir2.1-mediated resting membrane potential, Cx43-mediated cell-to-cell conduction, and HCN4-dependent pacemaker current. miR-1 is relevant because it increases in ischemic myocardium and can repress *KCNJ2*/Kir2.1 and *GJA1*/Cx43, linking a molecular perturbation to slowed conduction and arrhythmia susceptibility. Associations between miR-1/miR-133 and HCN-channel expression have also been reported in atrial-fibrillation tissue. Direct causal support is therefore stronger for the Kir2.1/Cx43 axis than for HCN4 regulation [90,91].

#### 4.4.2. Atrial Fibrillation: Direct Evidence for TCONS_00075467 and NEAT1, and Exclusion of Unsupported HOTAIR Claims

In a rabbit atrial-fibrillation model, TCONS_00075467 perturbation supported a miR-328-*CACNA1C* axis and altered L-type-calcium-current-related electrical remodeling; human orthology and clinical target engagement remain unknown. NEAT1 has a different, structural endpoint: it was increased in atrial tissue from patients with atrial fibrillation and, in angiotensin-II-treated fibroblasts and mice, promoted proliferation, migration, collagen production, and atrial fibrosis through a proposed miR-320-NPAS2 competing-RNA pathway. The cited HOTAIR item is a one-page journal Comment with no abstract or original experimental data. It cannot support a myocardial-fibrosis mechanism or an atrial-fibrillation claim and is retained only to make the evidentiary exclusion transparent [92,93,94].

#### 4.4.3. Ventricular Arrhythmia After MI: Scar-Border-Zone ncRNA Dysregulation

The infarct border zone combines heterogeneous conduction, altered repolarization, calcium instability, and fibrosis. Repression of Kir2.1 and Cx43 by miR-1 links an ncRNA directly to post-MI ventricular arrhythmia susceptibility [90]. Other profiling studies have produced candidate signatures, but many lack cell-type resolution and prospective arrhythmia endpoints. Mechanistic claims should require electrophysiology, target rescue, and arrhythmia testing rather than expression differences alone.

#### 4.4.4. Inherited Channelopathies: ncRNA-Related Variants as Penetrance Modifiers

Noncoding variants can modify channel expression by altering miRNA binding or RNA stability, but convincing human evidence is limited. *KCNQ1* 3′-UTR variants were initially associated with allele-specific expression and phenotype severity in long-QT syndrome [95]; a larger analysis did not confirm an independent modifying effect after accounting for founder mutations and population structure [96]. For Brugada syndrome, candidate SCN5A noncoding variants remain biologically plausible but are not validated clinical penetrance modifiers. Such variants should be classified with family segregation, functional assays, and replication rather than computational prediction alone.

### 4.5. Hypertension and Vascular Remodeling—ncRNAs in Pressure-Overload Adaptation

Hypertension integrates renal sodium handling, the renin–angiotensin–aldosterone system (RAAS), neurohumoral activation, endothelial dysfunction, and arterial remodeling. ncRNA studies often capture one compartment at a time; causal interpretation therefore requires matching the experimental tissue and disease model to the proposed systemic mechanism.

#### 4.5.1. Hypertensive VSMC Remodeling: EV miR-21-3p and AK098656

Two studies directly connect ncRNAs to VSMC state in hypertension. In spontaneously hypertensive rats, adventitial-fibroblast EVs transferred miR-21-3p to VSMCs and increased proliferation and migration, although absolute vesicular copies and in vivo recipient-cell target engagement were not established. AK098656 was VSMC-dominant in human profiling; gain- and loss-of-function experiments supported a shift toward a synthetic phenotype and a contribution to hypertension in animal models. The originally listed miR-155 and H19 examples were removed from this subsection because the cited studies did not provide comparable hypertension-specific VSMC causality [97,98].

#### 4.5.2. Renal ncRNAs as Bounded Upstream Modifiers of RAAS and Cardiac Afterload

The renal examples are included for a specific cardiovascular reason: renin output and angiotensin-II signaling determine vascular resistance, volume state, and therefore myocardial afterload. A common *AGTR1* 3′-UTR variant modifies miR-155-mediated repression of the angiotensin-II type-1 receptor, while renal miR-181a represses renin and its deficiency increases blood pressure in mice. These studies support compartment-specific regulation of an upstream cardiac stressor. They do not justify extrapolating the review to kidney disease generally or treating circulating ncRNA abundance as a direct measure of intrarenal RAAS activity [74,75,76].

#### 4.5.3. Endothelial Dysfunction Under Chronic Pressure: MIAT and NO Bioavailability

MIAT can modulate endothelial survival, inflammation, and microvascular responses in diabetic and ischemic settings, but direct evidence that MIAT controls nitric-oxide bioavailability during chronic systemic hypertension is insufficient. The stronger endothelial-NO evidence concerns LEENE and STEEL [13,79]. MIAT may be discussed as a candidate stress-response lncRNA, not as an established hypertension-specific NO regulator, until endothelial-restricted perturbation is tested in validated pressure models.

#### 4.5.4. Pulmonary Arterial Hypertension as a Vascular–Right-Ventricular Coupling Model

Pulmonary arterial hypertension (PAH) is included because it couples a defined vascular lesion to progressive right-ventricular pressure overload. miR-145 has experimental and patient-sample support in pulmonary vascular remodeling, whereas H19 has been linked to decompensated right-ventricular failure. This pairing allows vascular and myocardial compartments to be evaluated within one disease trajectory. The pulmonary and systemic circulations are not treated as interchangeable, and PAH is not presented as evidence that every pulmonary ncRNA mechanism belongs within a cardiac review [77,78].

### 4.6. Diabetic Cardiomyopathy—Metabolic Reprogramming Through ncRNA Networks

Diabetic cardiomyopathy emerges from chronic substrate excess, oxidative stress, mitochondrial dysfunction, impaired autophagy, cell death, fibrosis, and microvascular injury. Because diabetes affects many organs and blood-cell populations, cardiac tissue evidence should be distinguished from circulating associations and from mechanisms established only in vascular or pancreatic cells.

#### 4.6.1. Hyperglycemic Stress: Cell-Specific miR-34a Evidence and the miR-200 Boundary

Human diabetic myocardium, adult human cardiomyocytes exposed to high glucose, and diabetic-heart progenitor cells showed increased miR-34a with lower SIRT1. miR-34a inhibition reduced high-glucose-induced apoptosis in cardiomyocytes but did not rescue progenitor-cell proliferation, directly demonstrating that the same intervention has cell-dependent consequences. In diabetic mice subjected to subsequent glycemic normalization, persistent miR-34a and miR-218 dysregulation was linked to DNMT3b-SIRT1 control of the pro-oxidant adaptor p66Shc and residual ventricular dysfunction. By contrast, the cited set does not provide comparable cardiomyocyte-restricted causal evidence for the miR-200 family, so miR-200 is treated as a hyperglycemic-stress candidate rather than a validated master regulator of diabetic cardiomyopathy [99,100].

#### 4.6.2. ZFAS1 and Ferroptosis: In Vivo Phenotype Versus ceRNA Stoichiometry

ZFAS1 was tested in high-glucose-treated cardiomyocytes and db/db mice using gain- and loss-of-function constructs. Its inhibition reduced collagen deposition, apoptosis, ferroptosis-associated readouts, and ventricular injury, and miR-150-5p/CCND2 perturbation provided pathway rescue. This supports a preclinical cardiac phenotype. The proposed competing-endogenous-RNA mechanism still requires absolute cytoplasmic ZFAS1 and miR-150-5p abundance, accessible site number, and endogenous AGO occupancy to show that physiologic competition is quantitatively plausible. MEG3 was removed from this subsection because the cited reference set did not directly support the claimed interference with cardiac insulin signaling or lipotoxic injury [101].

#### 4.6.3. circMAP3K5 Addresses Apoptosis, Whereas H19 Addresses Autophagy

circMAP3K5 was identified by profiling a small set of diabetic-rat hearts, but its functional depletion and miR-22-3p/DAPK2 rescue experiments were performed mainly in high-glucose-treated H9c2 cardiomyoblasts and measured apoptosis. It therefore supports a candidate cell-death pathway, not an in vivo circRNA mechanism of defective autophagy flux. H19, by contrast, was manipulated in diabetic rats and neonatal cardiomyocytes and linked through EZH2/H3K27me3 to DIRAS3-mTOR-associated autophagy and ventricular function. Even for H19, static autophagy markers should not be equated with complete lysosomal flux unless flux is directly measured. The two RNAs are retained as examples of distinct endpoints rather than one circRNA-autophagy module [102,103].

#### 4.6.4. Epigenetic Memory After Glycemic Normalization

Experimental diabetes leaves a persistent cardiac ncRNA signature after normoglycemia is restored. Hundreds of miRNAs remained altered in diabetic mouse hearts, supporting a molecular memory of prior hyperglycemia [104]. Subsequent work linked persistent miR-34a/miR-218 dysregulation to DNMT3b, SIRT1, and p66Shc, coupling ncRNA regulation to chromatin and oxidative stress [100]. Whether these signatures predict residual cardiovascular risk in intensively treated patients remains to be established prospectively.

### 4.7. Cardiac Aging—The ncRNA Epigenetic Clock and Senescence Enforcement

Cardiac aging combines cardiomyocyte loss, hypertrophy, mitochondrial decline, fibrosis, vascular dysfunction, immune activation, and reduced stress tolerance. ncRNAs contribute to several of these processes, but the phrase “ncRNA epigenetic clock” should not imply the existence of a validated cardiac age estimator comparable to DNA-methylation clocks.

#### 4.7.1. Direct Cardiac-Aging Evidence: miR-34a and the miR-29 Family

In aged mouse hearts, miR-34a represses PNUTS, a phosphatase-regulatory protein involved in DNA-damage control, and promotes telomere damage, cardiomyocyte death, and functional decline; inhibition improves recovery after injury. The miR-29 family participates in age-dependent extracellular-matrix and chromatin regulation, including a TGF-β-miR-29-H4K20me3 axis. These studies provide cardiac perturbation evidence, while also showing why therapeutic direction depends on cell type: miR-29 can restrain fibrosis yet alter genome-maintenance pathways [105,106].

#### 4.7.2. Evidence Boundaries for Candidate Cardiac-Aging ncRNAs

TERRA, NEAT1, SncmtRNA/ASncmtRNA, senescence-associated lncRNAs, and young-plasma ncRNAs remain candidate links to cardiac aging rather than established mechanisms. Telomere damage is documented in cardiac disease, but a causal TERRA pathway in aged cardiomyocytes has not been shown. Mitochondrial ncRNAs and SASP-regulating lncRNAs are supported mainly by noncardiac or descriptive evidence. Heterochronic-parabiosis studies of GDF11 were not reproducible, and no validated GDF11-ncRNA rejuvenation axis has emerged. These candidates are retained only to define experiments that are still needed: cardiac-cell localization, physiological abundance, age-specific perturbation, target rescue, and organ-level functional testing [15,107,108,109].

### 4.8. Cross-Disease Synthesis—Shared Modules, Conditional Effects, and Biomarker Limits

Across the selected diseases, convergence occurs at the level of regulatory modules rather than at the level of a uniformly acting “pan-CVD” RNA. Endothelial stress and vascular-state transitions recur in atherosclerosis and hypertension; innate immune and matrix programs recur after infarction and in heart failure; survival and mitochondrial stress recur in ischemia, diabetes, and aging; and electrical remodeling links hypertrophy, scar, and arrhythmia. miR-21, MALAT1, NEAT1, and H19 are informative because they occupy more than one module, but their broad expression also makes direction of effect and systemic safety context-dependent [15,110,111].

#### 4.8.1. From Recurrent ncRNAs to Cell-State Modules

A credible cross-disease network should integrate cell-resolved expression, experimentally supported RNA–target edges, disease phase, and direction of effect. Simple overlap of differentially expressed ncRNAs is insufficient because the same signal may reflect altered cell composition, medication, or injury severity. Recurrent RNAs are most useful for identifying modules—fibrosis, immune resolution, endothelial dysfunction, mitochondrial stress, or conduction—not for assuming one therapeutic direction across diagnoses [15,85,86,110,111].

#### 4.8.2. Why Direction of Effect Changes with Cell Type and Disease Phase

Apparent paradoxes are expected when an ncRNA targets multiple transcripts in different cells. miR-21 can promote cardiomyocyte survival acutely yet support fibroblast or macrophage programs that worsen later remodeling [85,86]. miR-155 likewise changes function with lesion stage and immune state [60,61]. Resolution requires cell-specific perturbation, time-course experiments, target rescue, and clinically relevant comorbid models rather than assigning a fixed label of protective or pathogenic.

#### 4.8.3. What Shared ncRNA Signals Can—And Cannot—Do as Biomarkers

A shared circulating ncRNA panel could capture cumulative cardiovascular stress, but existing studies are diagnosis-specific case-control or prognostic cohorts rather than a validated pan-cardiovascular test. Coronary-disease miRNA profiles, LIPCAR, miR-423-5p, and DCM-associated circRNAs differ in source population, specimen processing, normalization, and endpoint. None localizes disease by itself. Development therefore requires locked pre-analytics, hemolysis and platelet controls, renal-function and medication adjustment, prospective external validation, and incremental value beyond troponin, natriuretic peptides, imaging, and established risk scores [66,67,70,72,73]. The disease-specific evidence discussed above is summarized in Table 3.

## 5. Discussion and Future Perspectives

The principal gap is not a shortage of ncRNA catalogues but inconsistent linkage between a molecule, the cardiovascular process it is proposed to regulate, and the model that generated the evidence. We therefore interpret every example through four questions: What biological transition failed or was restored? Which cell and species were tested? What result was directly observed? What inference remains after model and endpoint limitations are considered? Across development, regeneration, and disease, ncRNAs can change chromatin competence, target-network output, intercellular signaling, or response duration, but the same transcript may be adaptive in one cell and phase and harmful in another. An expression change alone is not a mechanism [2,3,7,8,9,10,11,12,13,14,15,16,17,18,19,20,21,22,23,24,37].

**Figure 4:**
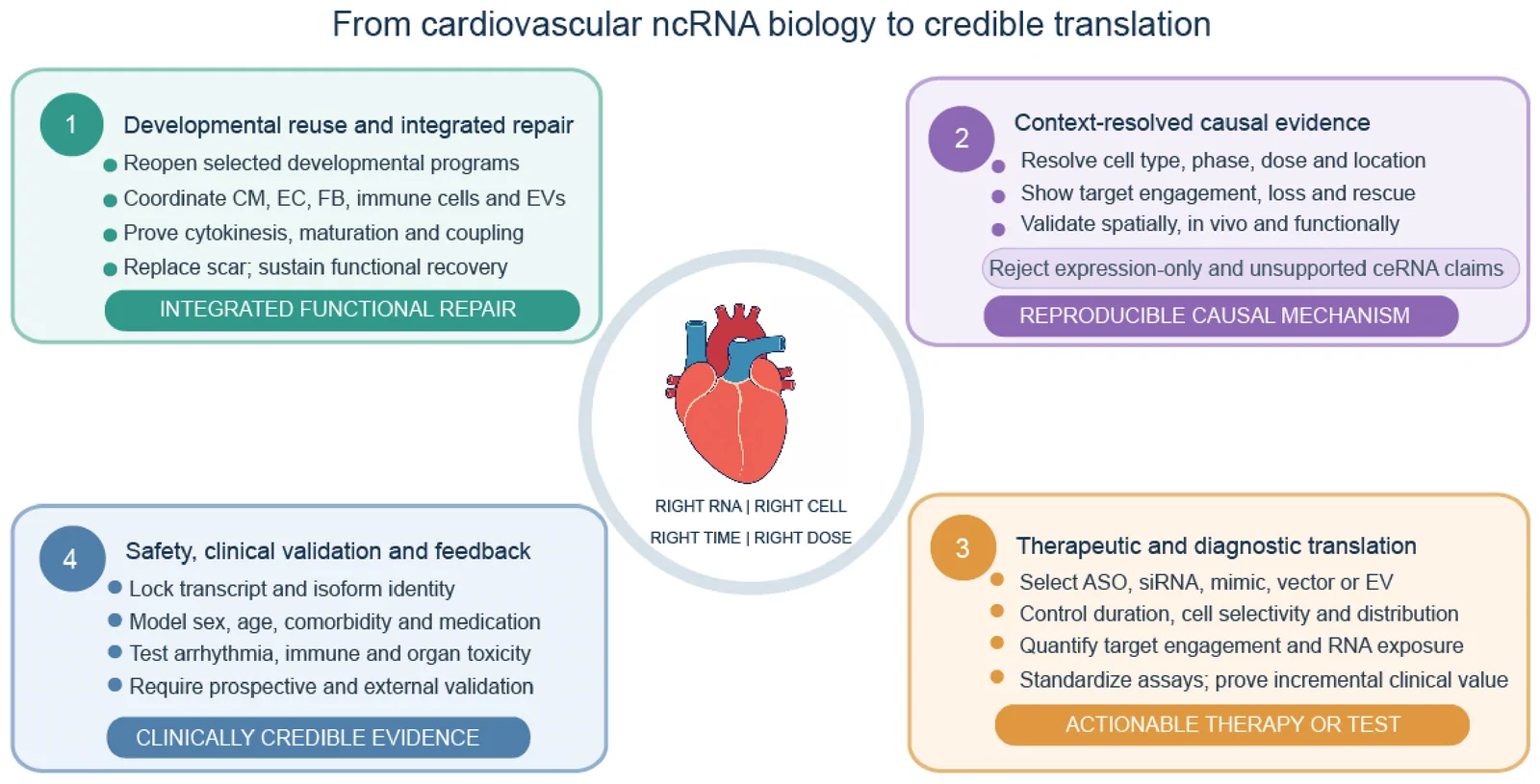
Framework for translating cardiovascular ncRNA biology into clinical applications. Successful translation requires integrated repair, context-resolved causal evidence, targeted therapeutic or diagnostic strategies, and rigorous safety and clinical validation, guided by the right RNA, cell, time, and dose. Abbreviations: ASO, antisense oligonucleotide; CM, cardiomyocyte; EC, endothelial cell; EV, extracellular vesicle; FB, fibroblast; siRNA, small interfering RNA.

This review consequently proposes a four-coordinate interpretation for every ncRNA claim: cell identity, temporal phase, physiological target engagement, and tissue consequence. Development defines the intended trajectory; injury tests whether part of that trajectory can be reopened safely; disease reveals how the same modules become chronically or ectopically engaged. Vascular, renal-RAAS, and pulmonary examples are retained only when they change perfusion or load, reproduce one of these modules, and culminate in a cardiac endpoint. This framework is the conceptual link among Section 2, Section 3 and Section 4 and the basis for the translational filter in Figure 4 [2,3,21,37,74,75,76,77,78].

### 5.1. Developmental Regulatory Grammar Is Reused—But Not Fully Recreated—During Repair

Developmental ncRNAs cooperate with chromatin regulators and transcription factors to create stable but adaptable cell states. FENDRR, CARMEN, and Braveheart illustrate how lncRNAs participate in lineage specification, while miR-1 and miR-133 refine cardiomyocyte proliferation, differentiation, and electrical maturation [7,8,9,10,12]. Regenerative strategies reactivate only part of this grammar. miR-199a and the miR-302/367 cluster can increase cardiomyocyte cell-cycle activity through coordinated repression of Hippo–YAP pathway components [20,40], but cell-cycle markers do not prove production of mature, force-generating, electrically integrated cardiomyocytes. The lethal arrhythmias caused by persistent miR-199a expression in pigs demonstrate that proliferative potency can outpace tissue-level maturation [21]. Regeneration should therefore be evaluated using lineage tracing, cytokinesis, ploidy, sarcomere maturation, electromechanical coupling, arrhythmia monitoring, scar replacement, and durable functional recovery.

### 5.2. Cardiac Repair Is a Multicellular and Inter-Organ Communication Problem

Cardiomyocyte-centered approaches cannot fully account for vascular supply, extracellular-matrix organization, immune resolution, or electrophysiological integration. Endothelial ncRNAs such as STEEL and LEENE regulate flow-responsive and nitric-oxide-associated programs [13,79]; Wisper and NEAT1 influence fibroblast activation and matrix deposition [14,15]; and macrophage miR-155/miR-146a circuits shape the amplitude and resolution of innate immune signaling [55,56]. Extracellular vesicles add a transferable layer through which progenitor-like cells, cardiomyocytes, endothelium, and macrophages can exchange regulatory cargo [23,24,47]. However, evidence that an EV preparation is protective does not establish that a specific RNA is necessary or sufficient. Cargo-specific claims require depletion and rescue experiments, physiologically plausible RNA copy numbers, recipient-cell target engagement, and rigorous characterization of vesicle identity and biodistribution.

### 5.3. Pleiotropy and Context Dependence Are Core Biology, Not Experimental Noise

The same ncRNA can produce opposite outcomes because its accessible targets differ across cells and phases. miR-21 replacement reduced apoptosis in a rat-heart/H9c2 model of acute ischemia-reperfusion, whereas delayed intracoronary anti-miR-21 in pigs reduced macrophage- and fibroblast-associated chronic remodeling after ischemia-reperfusion. These experiments address different time windows and compartments and are not a simple contradiction. miR-155 likewise changes function with atherosclerotic lesion stage, while MALAT1, NEAT1, and H19 act in several endothelial, fibroblast, cardiomyocyte, and immune contexts. Therapeutic direction must therefore be derived from cell-resolved target engagement and exposure timing rather than a fixed protective or pathogenic label [15,60,61,85,86,110,111].

### 5.4. Mechanistic Claims Require a More Explicit Evidence Hierarchy

Much of the cardiovascular ncRNA literature begins with differential expression, then moves to reporter assays and overexpression or knockdown in immortalized cells. The sequence is useful for hypothesis generation, but it often overstates causality. A mechanistic claim that can withstand replication should connect endogenous transcript abundance with target occupancy, include loss-of-function and rescue, establish the relevant subcellular localization, and reproduce the phenotype in an appropriate in vivo model. The bar is especially important for competing-endogenous-RNA models, which require enough ncRNA and miRNA-binding sites to perturb the shared target pool. A circRNA should likewise not be assigned a sponge or protein-scaffold function from sequence prediction or pulldown data alone.

We therefore apply an explicit evidence ladder. Tier 1 comprises bulk or circulating differential expression and is hypothesis-generating. Tier 2 adds endogenous localization, physiological abundance, reporter or biochemical interaction, and primary-cell perturbation. Tier 3 requires cell-restricted in vivo loss-of-function, target rescue, and a disease-relevant phenotype. Tier 4 adds large-animal testing or human myocardial target engagement, exposure-response analysis, independent replication, and clinically meaningful safety and function. For EV-RNA mechanisms, particle identity, recovery and purity, absolute cargo copies, cargo-specific depletion/reconstitution, recipient-cell delivery, and target repression are additional requirements. For regeneration, even Tier 3 molecular causality does not substitute for the tissue-level endpoints defined in Section 3 [2,3,37].

The primary-study audit changed several conclusions rather than merely adding caveats. The foam-cell transcript is lincRNA-DYNLRB2-2, not DYNAMO. HRCR has direct hypertrophy evidence, not a proven ischemia-reperfusion ROS-buffering role. The HOTAIR citation is a Comment without original data and cannot support atrial-fibrillation or myocardial-fibrosis causality. circMAP3K5 functional evidence is mainly an H9c2 apoptosis experiment, not in vivo autophagy-flux control. The proposed GDF11 cardiac-rejuvenation phenotype was not independently reproduced. These examples show why publication type, experimental model, measured endpoint, and contradictory evidence must be checked before a molecular association is incorporated into a cardiovascular mechanism [62,84,93,102,108,109].

### 5.5. Therapeutic Translation Requires Transient, Cell-Selective, and Measurable Target Engagement

Antisense oligonucleotides, siRNAs, miRNA mimics, viral vectors, nanoparticles, and engineered EVs offer distinct combinations of duration, tissue exposure, and manufacturability. Short-lived oligonucleotide delivery may be appropriate for acute reperfusion or temporary cell-cycle induction, whereas chronic heart failure and vascular remodeling may require repeated dosing. Nuclear lncRNAs may be accessible to antisense strategies but difficult to replace, whereas miRNA mimics can simultaneously influence many unintended targets. Preclinical success with anti-miR-15, Chast inhibition, and Wisper silencing demonstrates therapeutic feasibility [14,68,69], but the miR-199a pig study defines the safety standard for regenerative approaches [21]. Future studies should quantify RNA exposure and target engagement in each cardiac cell type, incorporate clinically relevant comorbidities and background therapy, and assess arrhythmia, immune activation, hepatic and renal toxicity, and durability of benefit.

Regenerative translation faces additional hurdles beyond molecular efficacy. Adult human cardiomyocytes differ from neonatal rodents in ploidy, metabolic state, cell-cycle competence, size, and conduction reserve; the dose that induces transient cycling in mice may produce incomplete division or electrical instability in a large adult heart. Delivery must reach the intended cardiac cell population while limiting liver, kidney, vascular, and immune exposure; expression must switch off after cytokinesis; and newly formed cells must mature within a fibrotic and mechanically loaded border zone. Manufacturing consistency, arrhythmia monitoring, long-term tumor and immune surveillance, and efficacy on top of reperfusion and guideline-directed therapy are therefore integral to, rather than downstream of, proof of regeneration [21,37].

### 5.6. Current Human Clinical Trials of Cardiovascular ncRNA Therapeutics

Human testing remains limited but now provides a direct test of whether pharmacological target engagement translates into cardiac benefit. CDR132L is an antisense oligonucleotide that inhibits miR-132-3p. In a randomized phase 1b study of 28 patients with stable ischemic heart failure receiving standard care, two intravenous doses were well tolerated and produced dose-dependent, sustained plasma miR-132 reduction; exploratory changes in NT-proBNP and QRS duration were hypothesis-generating because the trial was small and not powered for efficacy [4].

The subsequent multinational HF-REVERT phase 2 trial randomized 294 patients 3–14 days after myocardial infarction and with left ventricular ejection fraction of 45% or lower to CDR132L 5 mg/kg, 10 mg/kg, or placebo in addition to guideline-directed therapy. CDR132L was well tolerated and reduced circulating miR-132, but neither dose significantly improved the primary 6-month change in left ventricular end-systolic volume index or the principal secondary endpoints versus placebo. Exploratory subgroup signals do not establish efficacy. Two phase 2 studies in chronic heart failure with preserved ejection fraction or reduced/mildly reduced ejection fraction are recruiting (NCT06979362 and NCT06979375), and should test whether disease stage, remodeling burden, and treatment duration alter response [5].

MRG-110, a locked-nucleic-acid anti-miR-92a, provides a different level of clinical evidence. A first-in-human, single-dose escalation study in healthy adults demonstrated dose-dependent suppression of miR-92a in blood and CD31+ cells and derepression of ITGA5 and CD93. However, no patients with cardiac dysfunction were treated and no cardiovascular efficacy endpoint was tested; MRG-110 should therefore be described as evidence of systemic pharmacology and target engagement, not as a proven cardiac therapy. Together, these programs show that measurable ncRNA engagement is feasible in humans, but clinical translation still requires myocardial or disease-compartment exposure, relevant functional endpoints, adequate representation of women, and incremental benefit over contemporary therapy [6].

### 5.7. Circulating ncRNAs Are Promising Risk Signals but Remain Immature Clinical Tests

Circulating miRNAs, lncRNAs, and circRNAs are attractive because blood sampling is scalable and repeatable. Candidate signals have been reported for coronary artery disease, ventricular remodeling, heart failure, and dilated cardiomyopathy, including vascular miRNA profiles, miR-423-5p, LIPCAR, and selected circRNAs [66,70,72,73]. Yet analytical and biological confounding remains substantial. Hemolysis, platelet activation, leukocyte composition, renal clearance, medication, exercise, sampling time, extraction method, and normalization strategy can all change measured ncRNA abundance. A clinically useful assay must have locked pre-analytical procedures, analytical quality controls, external validation, and demonstrated incremental value beyond established biomarkers, imaging, and risk scores. Broad “pan-CVD” panels may capture cumulative cardiovascular stress, but they are unlikely to localize disease without complementary clinical information.

### 5.8. Priorities for the Next Generation of Cardiovascular ncRNA Research

Several practical changes would make the next generation of studies more informative. Transcript nomenclature and isoform identity need to be unambiguous, particularly for lncRNAs and circRNAs. Cell-resolved and spatial measurements should precede therapeutic design, and perturbations should approximate endogenous rather than supraphysiological expression. Developmental, injury, and disease models also need to account for sex, age, metabolic status, rhythm, and common cardiovascular medications. When proliferation, conduction, biodistribution, or device-compatible delivery is central to the hypothesis, large-animal testing should begin earlier. EV studies require comparable isolation methods, cargo quantification, potency assays, and manufacturing criteria. Biomarker studies, in turn, should be prospective, adequately powered, and registered with prespecified endpoints and analysis plans.

### 5.9. Conclusions

ncRNAs are best understood as context-dependent regulators of cardiovascular cell-state transitions, not as isolated switches assigned permanently to one disease. Cardiac development supplies the reference sequence of lineage commitment, morphogenesis, and maturation; regenerative experiments test whether selected transitions can be reopened; and cardiovascular disease shows how the same modules are mis-timed, prolonged, or activated in the wrong compartment. The central translational requirement is therefore alignment of the right RNA with the right cell, phase, endogenous molecular interaction, and tissue endpoint. With these coordinates made explicit—and with vascular or systemic examples restricted to direct cardiac consequences—ncRNA biology offers a coherent framework for mechanism, regeneration, biomarkers, and therapy without overstating the evidence [2,3,21,37].

## Figures and Tables

**Table 1:** Model-resolved evidence for ncRNAs in cardiac development and differentiation.

Reference	ncRNA/Axis	Study Model or Evidence	Main Finding
Grote, 2013 [7]	FENDRR-PRC2/TrxG	*Fendrr*-null mouse embryos; chromatin analyses	FENDRR regulates activating and repressive chromatin marks at lateral-plate-mesoderm and cardiac transcription-factor loci and is required for cardiac and body-wall development.
Ounzain, 2015 [8]	CARMEN-PRC2	Human fetal cardiac progenitor cells; knockdown and differentiation	CARMEN is a super-enhancer-associated lncRNA required for cardiac specification and differentiation.
Plaisance, 2023 [25]	CARMEN-201-REST	Human cardiac precursor cells; isoform-specific functional analysis	A MIRc-containing CARMEN isoform recruits REST and shifts precursor-cell specification from cardiomyocyte toward smooth-muscle fate.
Liu, 2021 [26]	HBL1-PRC2	Human pluripotent stem cells; ChIP-seq and loss-of-function	HBL1 is required for genome-wide PRC2 occupancy and proper cardiogenic differentiation.
Zhao, 2005 [10]	miR-1-*Hand2*	Cardiac miR-1 gain-of-function mouse embryos	SRF-regulated miR-1 targets *Hand2* and limits ventricular cardiomyocyte expansion during cardiogenesis.
Heidersbach, 2013 [11]	miR-1 dosage	Compound miR-1-1/miR-1-2 knockout mice	Combined miR-1 loss causes embryonic lethality and sarcomere disruption, demonstrating dosage dependence.
Liu, 2008 [12]	miR-133a	miR-133a-1/miR-133a-2 double-knockout mice	miR-133a controls cardiomyocyte proliferation, survival, and repression of smooth-muscle genes.
Callis, 2009 [28]	miR-208a-Thrap1/GATA4/Hop/Cx40	miR-208a transgenic and knockout mice	miR-208a links myosin expression to hypertrophy and cardiac-conduction programs.
Klattenhoff, 2013 [9]	Braveheart-PRC2	Mouse embryonic-stem-cell cardiac differentiation	Braveheart acts upstream of MesP1 and is required for cardiovascular lineage commitment.
Man, 2018 [13]	STEEL-eNOS/KLF2	Human endothelial cells under static and flow conditions	STEEL maintains basal endothelial transcriptional programs and participates in flow-responsive angiogenic patterning.
Zhang, 2013 [29]	miR-126-PIK3R2/PI3K/Akt	Endothelial-progenitor-cell EndMT model	miR-126 inhibits endothelial-to-mesenchymal transition through PIK3R2-PI3K/Akt signaling.
Lagendijk, 2011 [30]	miR-23-*Has2*	Zebrafish cardiac-valve development	miR-23 limits valve formation by repressing *Has2* and extracellular hyaluronan production.
Chapnik, 2012 [32]	DGCR8-miRNA biogenesis	Neural-crest-specific *Dgcr8* conditional knockout mice	DGCR8-dependent miRNA processing is required for cardiac neural-crest survival and outflow-tract development.
Racedo, 2024 [33]	DGCR8 in the secondary heart field	Secondary-heart-field *Dgcr8* conditional knockout mice	DGCR8 is required in the secondary heart field for outflow-tract and right-ventricular development.

**Table 2:** Cell-, species-, and endpoint-resolved evidence for ncRNAs in cardiac regeneration.

Reference	ncRNA/Axis	Cell Type, Species, and Human Validation	Endpoint, Evidence Strength, and Principal Limitation
Bergmann, 2009 [16]	Adult cardiomyocyte renewal	Human cardiomyocyte nuclei; retrospective 14C birth dating; direct human evidence.	Quantifies low age-dependent renewal, but does not identify an injury mechanism or demonstrate regenerative remuscularization.
Porrello, 2011 [18]	Postnatal regenerative window	Neonatal mouse cardiomyocytes after apical resection; no human validation.	Supports a brief scar-free regenerative window; sensitivity to injury model and neonatal stage limits extrapolation to adult human MI.
Senyo, 2013 [17]	Source of new cardiomyocytes	Adult mouse pre-existing cardiomyocytes; lineage tracing and isotope imaging; complementary human turnover context only.	Strong source-mapping evidence for low-level cardiomyocyte renewal, but not a therapeutic regeneration experiment.
Xin, 2013 [38]	YAP/TAZ	Cardiomyocyte-restricted YAP/TAZ mouse models; no direct human validation.	Genetic causality and functional repair are strong; daughter-cell tracing, long-term maturation, and electrical integration remain incomplete.
Heallen, 2013 [39]	Hippo–YAP	Postnatal and adult mouse cardiomyocytes with cardiac Hippo deficiency; no human validation.	Supports Hippo restraint of cytokinesis and repair; supraphysiological pathway release and incomplete integration endpoints limit translation.
Eulalio, 2012 [19]	miR-199a-3p/miR-590-3p	Neonatal rat/mouse cardiomyocytes and mouse MI; no human validation.	DNA synthesis, mitotic/cytokinetic markers, and function support proliferation, but clonal lineage, net cardiomyocyte number, and electrical integration were not established.
Torrini, 2019 [40]	miR-199a–TAOK1/β-TrCP/Cofilin2–YAP	Predominantly neonatal rodent cardiomyocytes with target validation; no human injury validation.	Strong multi-target Hippo-YAP mechanism; mainly cell and marker endpoints, not tissue-level regeneration.
Gabisonia, 2019 [21]	AAV6–miR-199a	Adult porcine cardiomyocytes after MI; translational large-animal model; no human study.	Early scar and functional improvement were followed by lethal ventricular arrhythmias; persistent AAV exposure failed the durability and safety criteria for true regeneration.
Tian, 2015 [20]	miR-302/367–Mst1/Lats2/Mob1b	Genetic mouse cardiomyocyte models and transient miRNA mimics; no direct human validation.	Transient treatment improved repair whereas sustained expression caused dedifferentiation and dysfunction; lineage and electrical-integration endpoints were incomplete.
Cai, 2018 [41]	CAREL–miR-296–Trp53inp1/Itm2a	Neonatal and adult mouse cardiomyocytes; transgenic and viral perturbation; no human validation.	In vivo division and repair phenotype is persuasive, but the CAREL-miR-296 ceRNA mechanism lacks complete endogenous stoichiometric validation.
Han, 2014 [42]	Mhrt–BRG1	Adult mouse cardiomyocytes under pressure overload; no direct human functional validation.	Strong Mhrt-BRG1 chromatin and cardioprotection evidence, but no lineage tracing, cytokinesis, or myocardial regeneration endpoint.
Li, 2025 [43]	LncBAR–BRG1	Neonatal/adult mouse cardiomyocytes and injury models; complementary hESC-cardiomyocyte data.	Gain- and loss-of-function support LncBAR-BRG1 control of cycling and repair; human injured myocardium, maturation, and electrical integration remain untested.
Ruberto, 2026 [44]	CLIPPER–LPIN1	Mouse cardiomyocyte screen and MI; conserved human locus/function in cultured human systems.	CLIPPER-LPIN1 target engagement links mitochondrial remodeling to proliferation; clinical myocardial validation and durable safety are absent.
Chen, 2013 [45]	CPC-EVs/miR-451	Mouse CPC preparation to H9C2 cardiomyoblasts and mouse ischemia/reperfusion; no human validation.	Electron microscopy/CD63, uptake, and cytoprotection were shown; miR-451 necessity, recovery/purity, absolute copy number, and recipient target engagement were not established.
Barile, 2014 [22]	CPC-EV miR-210/miR-132	Human atrial CPC-EVs to mouse HL-1 cardiomyocytic cells, human endothelial cells, and mouse MI.	EV depletion, donor-cell control, and miR-210/miR-132 target repression support cardioprotection/angiogenesis; in vivo cell-specific uptake and RNA copies were not quantified.
Ibrahim, 2014 [23]	CDC-exosomal miR-146a	Human CDC-derived exosomes, cardiomyocyte/endothelial assays, and mouse MI; no clinical validation.	Blocking exosome production and partial phenocopy by miR-146a support multi-cargo activity; native miR-146a dose, lineage tracing, and electrical integration were not resolved.
Xiao, 2016 [46]	CPC-exosomal miR-21–PDCD4	Mouse CPC-EVs to H9C2 cardiomyoblasts; limited in vivo evidence; no human validation.	Particle characterization and miR-21-PDCD4 repression support cytoprotection; primary adult cardiomyocytes, absolute RNA copies, and regeneration endpoints are lacking.
Cambier, 2017 [47]	EV-YF1–IL-10	Human CDC-EVs to macrophages, rat cardiomyocytes, and rodent ischemia/reperfusion; no clinical study.	Abundant EV-YF1 transfer and IL-10 induction support macrophage-mediated cardioprotection; endogenous dose and new-cardiomyocyte formation were not demonstrated.
de Couto, 2017 [24]	CDC-EV miR-181b–PKCδ	Human CDC exosomes, rat and pig ischemia/reperfusion, and macrophage transfer.	miR-181b loading, macrophage transcriptomics, PKC-delta suppression, and adoptive transfer form a strong causal chain; absolute cargo dose, biodistribution, and durable regeneration remain unresolved.
Aurora, 2014 [48]	Regenerative macrophages	Neonatal mouse macrophages; depletion model; no human validation.	Shows macrophages are required for neonatal neoangiogenesis and repair, but does not identify a specific ncRNA mechanism.
Li, 2017 [49]	Mirt1-NF-κB	Mouse cardiac fibroblasts, adenoviral cardiac knockdown, and mouse infarction; no human validation.	Supports fibroblast-to-immune signaling, reduced infiltration, and repair; it does not establish a macrophage-autonomous lncRNA mechanism.
Chang, 2023 [50]	MALAT1-EZH2-PPAR-γ	Infarct-associated mouse macrophages, macrophage assays, and non-lineage-restricted in vivo inhibition; no clinical validation.	Biochemical interaction and inflammatory phenotypes support a macrophage-associated axis, but in vivo cell autonomy and composition effects remain unresolved.
Hu, 2016 [51]	lincRNA-Cox2–SWI/SNF	LPS-stimulated murine macrophages outside the cardiac setting.	Strong lincRNA-Cox2-SWI/SNF transcriptional mechanism, but no post-MI cell-specific validation.
Wan, 2013 [52]	MerTK-dependent efferocytosis	*Mertk*-deficient mouse macrophages after MI; no direct human validation.	Causal evidence for efferocytosis-dependent healing and function, but this is not itself an ncRNA or cardiomyocyte-regeneration mechanism.
Das, 2014 [53]	miR-21-PTEN/PDCD4	Human monocyte-derived macrophage efferocytosis in a non-cardiac wound-inflammation model.	Supports a post-efferocytosis anti-inflammatory program, but not cardiomyocyte efferocytosis or infarct repair in vivo.
Taganov, 2006 [54]	miR-146a–IRAK1/TRAF6	Activated human innate immune cells; non-cardiac context.	Direct IRAK1/TRAF6 targeting establishes negative feedback, but not post-injury cardiac efficacy.
Mann, 2017 [55]	miR-155/miR-146a–NF-κB network	Genetic mouse macrophage models; largely non-cardiac inflammatory context.	Defines miR-155/miR-146a kinetics and network behavior; translation to infarct-resident macrophage states requires testing.
Hu, 2019 [56]	miR-155-SOCS1/NF-κB	Systemic antagomir in mouse infarction plus BMDM-NRCM conditioned-medium assays; no human validation.	Reduced inflammation, ER stress, and apoptosis are supported; macrophage autonomy and myocardial regeneration are not.
He, 2024 [57]	M1-EV miR-155-IL-6R/JAK2/STAT3	Macrophage EVs, cultured cardiomyocytes, and peri-infarct lentiviral miR-155; no human validation.	Supports uptake, target engagement, and changes in Ki67/pH3; native-EV cargo necessity, cytokinesis, ploidy-normal daughters, and regeneration were not demonstrated.

**Table 3:** Key ncRNAs and supporting evidence across cardiovascular diseases.

Reference	ncRNA/Axis	Study Model or Evidence	Main Finding
Man, 2018 [13]	STEEL–KLF2/eNOS	Human endothelial cells; flow and loss-/gain-of-function studies	STEEL coordinates endothelial angiogenic and quiescence programs and is regulated by laminar flow.
Miao, 2018 [79]	LEENE–eNOS	Human endothelial cells and enhancer-associated RNA assays	LEENE promotes eNOS transcription and endothelial function.
Josipovic, 2018 [80]	LISPR1–S1PR1	Flow-exposed human endothelial cells	LISPR1 supports S1PR1 expression and endothelial signaling under laminar flow.
Rayner, 2011 [58]	miR-33–ABCA1	Mouse atherosclerosis and anti-miR treatment	miR-33 inhibition increases cholesterol efflux and promotes plaque regression.
Ouimet, 2017 [59]	miR-33 and macrophage metabolism	Macrophage and mouse atherosclerosis models	miR-33 coordinates cholesterol efflux with macrophage metabolic state.
Nazari-Jahantigh, 2012 [60]	miR-155–BCL6	Mouse and macrophage atherosclerosis models	miR-155 enhances inflammatory atherogenesis through BCL6 repression.
Wei, 2015 [61]	miR-155 stage dependence	Mouse atherosclerosis	miR-155 has lesion-stage-dependent effects through CSF1R- and BCL6-linked pathways.
Li, 2018 [62]	lincRNA-DYNLRB2-2–TLR2	THP-1/RAW264.7 foam cells and ApoE-deficient mice	DYNLRB2-2 promotes ABCA1-linked cholesterol efflux by reducing TLR2 expression; “DYNAMO” is not the verified transcript name.
Boettger, 2009 [63]	miR-143/145	Genetic mouse vascular models	Loss of miR-143/145 disrupts VSMC differentiation and vascular homeostasis.
Cordes, 2009 [64]	miR-143/145 transcriptional network	Murine progenitors, neural-crest-derived VSMCs, and fibroblast-to-VSMC reprogramming assays.	Supports smooth-muscle fate and contractile-state regulation; it does not by itself establish plaque stabilization.
Holdt, 2016 [65]	circANRIL–PES1	Human vascular cells and genetic association	circANRIL induces nucleolar stress and limits proliferation through impaired rRNA maturation.
Fichtlscherer, 2010 [66]	Circulating miRNA signature	Patients with coronary artery disease	Circulating endothelial- and vascular-associated miRNAs differ in coronary artery disease.
Ren, 2013 [67]	Circulating CAD miRNA panel	Clinical case–control cohorts	A plasma miRNA signature discriminated coronary artery disease but required broader validation.
Hullinger, 2012 [68]	miR-15 family inhibition	Mouse ischemic injury and anti-miR therapy	Anti-miR-15 reduces cardiomyocyte death, infarct size, and adverse remodeling.
Li, 2014 [81]	Circulating miR-144	Remote ischemic preconditioning models	miR-144 acts as a circulating component of remote cardioprotection.
Sun, 2019 [82]	MALAT1 and AKT-associated signaling	Whole rat myocardium with cardiac siRNA after I/R; expressing cell not identified.	Knockdown reduced infarction and apoptosis and restored AKT phosphorylation; direct MALAT1-AKT engagement and pathway necessity were not shown.
Chen, 2019 [83]	MIAT-NF-κB/PUMA	H9c2 hypoxia-reoxygenation and non-lineage-restricted lentiviral knockdown in mouse hearts.	Supports reduced apoptosis and NF-κB/PUMA-associated signaling; cell-specific in vivo causality is unresolved.
Wang, 2016 [84]	HRCR–miR-223	Mouse pressure-overload hypertrophy	HRCR suppresses hypertrophy by sequestering miR-223; it is not established as an I/R ROS buffer.
Yang, 2014 [85]	miR-21-PTEN/AKT	Rat-heart I/R and H9c2 hypoxia-reoxygenation models.	Supports acute anti-apoptotic signaling; cardiomyocyte-restricted in vivo target engagement was not established.
Hinkel, 2020 [86]	Delayed anti-miR-21 after I/R	Pigs treated intracoronarily on days 5 and 19 and assessed at day 33.	Reduced fibrosis, hypertrophy, macrophage/fibroblast abundance, and chronic dysfunction; this is post-injury remodeling, not acute reperfusion protection.
Qian, 2011 [87]	miR-24–BIM	Mouse MI and cardiomyocyte assays	miR-24 represses BIM and reduces ischemia-induced cardiomyocyte apoptosis.
Giricz, 2014 [89]	Vesicle-dependent transferable cardioprotection	Conditioned effluent from isolated preconditioned donor rat hearts transferred to isolated recipient hearts.	EV depletion abolished infarct-size protection; no inter-organ route or specific RNA cargo was established.
Viereck, 2016 [69]	Chast–PLEKHM1	Mouse pressure overload and antisense inhibition	Chast promotes hypertrophy; antisense targeting attenuates pathological remodeling.
Han, 2014 [42]	MHRT–BRG1	Mouse pressure overload and chromatin assays	MHRT blocks pathological BRG1-dependent chromatin reprogramming.
Kumarswamy, 2014 [70]	LIPCAR	Patients after MI and with heart failure	Circulating LIPCAR predicts remodeling and cardiovascular mortality.
Callis, 2009 [28]	miR-208a	Genetic mouse hypertrophy models	miR-208a controls stress-dependent hypertrophy and β-myosin-heavy-chain induction.
van Rooij, 2009 [71]	miR-208b/miR-499	Mouse and muscle gene-regulation studies	Myosin-embedded myomiRs reinforce slow-muscle and β-myosin programs.
Tijsen, 2010 [73]	Circulating miR-423-5p	Clinical heart-failure cohorts	miR-423-5p emerged as a candidate diagnostic biomarker for heart failure.
Costa, 2021 [72]	Circulating circRNAs	Multicenter DCM cohorts	Selected circRNAs showed potential for distinguishing DCM etiologies.
Yang, 2007 [90]	miR-1–*KCNJ2*/*GJA1*	Ischemic myocardium and arrhythmia models	miR-1 represses Kir2.1 and Cx43 and increases post-MI arrhythmia susceptibility.
Li, 2015 [91]	miR-1/133 and HCN expression	Human atrial tissue across age-associated AF	Expression was inversely associated, but the study did not establish direct causal targeting.
Li, 2017 [92]	TCONS_00075467–miR-328–*CACNA1C*	Rabbit atrial-fibrillation model	The lncRNA modulates an electrical-remodeling axis; human relevance remains unconfirmed.
Jiang, 2023 [93]	HOTAIR citation excluded	One-page journal Comment; no abstract or original experimental methods/results.	Cannot support myocardial-fibrosis or atrial-fibrillation causality and is not used as mechanistic evidence.
Dai, 2021 [94]	NEAT1-miR-320-NPAS2	Human AF atrial tissue association, Ang-II-treated fibroblasts, and a mouse atrial-fibrosis model.	Supports structural fibrotic remodeling through a proposed ceRNA axis; direct electrical remodeling and endogenous stoichiometry were not established.
Amin, 2012 [95]	*KCNQ1* 3′-UTR variants	Long-QT syndrome families and allele-specific assays	Noncoding variants were associated with allele-specific expression and phenotype severity.
Crotti, 2016 [96]	*KCNQ1* 3′-UTR modifier test	Expanded long-QT syndrome cohorts	The proposed modifier effect was not independently reproducible after population adjustment.
Zheng, 2023 [97]	EV miR-21-3p	Spontaneously hypertensive rats and VSMCs	Fibroblast EV miR-21-3p promotes VSMC proliferation in hypertension.
Jin, 2018 [98]	AK098656	Human association, VSMCs, and animal models	AK098656 promotes a synthetic VSMC phenotype and contributes to hypertension.
Sethupathy, 2007 [74]	miR-155–*AGTR1* 3′-UTR	Human genetic and reporter assays	*AGTR1* rs5186 alters miR-155-mediated receptor repression.
Marques, 2015 [75]	miR-181a–renin	Human kidney and molecular assays	miR-181a directly represses renin and is reduced in hypertensive kidneys.
Paterson, 2021 [76]	miR-181a and blood pressure	miR-181a-deficient mice	Loss of miR-181a increases renin-pathway activity and blood pressure.
Caruso, 2012 [77]	miR-145	Human and mouse pulmonary hypertension	miR-145 is increased in PAH and its inhibition protects experimental animals.
Omura, 2020 [78]	H19 and right-ventricular failure	Human PAH samples and preclinical models	H19 contributes to right-ventricular decompensation in PAH.
Fomison Nurse, 2018 [99]	miR-34a-SIRT1	Human diabetic myocardium, adult human cardiomyocytes, and diabetic cardiac progenitor cells.	Inhibition reduced cardiomyocyte apoptosis but not progenitor-cell dysfunction, demonstrating cell-specific therapeutic effects.
Ni, 2021 [101]	ZFAS1-miR-150-5p-CCND2	High-glucose cardiomyocytes and db/db mice with gain/loss and rescue experiments.	Supports a ferroptosis-associated cardiac phenotype; the ceRNA mechanism lacks complete endogenous abundance and AGO-occupancy validation.
Shen, 2024 [102]	circMAP3K5-miR-22-3p-DAPK2	Small diabetic-rat-heart profiling set and functional H9c2 high-glucose experiments.	Supports an apoptosis-associated candidate pathway; in vivo circRNA perturbation and autophagy-flux causality were not shown.
Zhuo, 2017 [103]	H19–DIRAS3 autophagy	Diabetic cardiomyopathy models	H19 regulates DIRAS3-dependent autophagy in diabetic cardiac injury.
Costantino, 2016 [104]	Persistent cardiac miRNA signature	Diabetic mice after glycemic normalization	Prior hyperglycemia leaves persistent cardiac miRNA dysregulation.
Costantino, 2018 [100]	miR-34a/218–DNMT3b–SIRT1–p66Shc	Diabetic epigenetic-memory models	Persistent miRNA changes connect chromatin regulation to oxidative stress after normoglycemia.
Boon, 2013 [105]	miR-34a–PNUTS	Aged mice and cardiomyocyte injury	miR-34a promotes telomere damage, cell death, and age-related functional decline.
Lyu, 2018 [106]	TGF-β–miR-29–H4K20me3	Mouse cardiac aging	miR-29 links extracellular signaling to age-associated chromatin changes.
Michalik, 2014 [110]	MALAT1 in endothelium	Human endothelial cells and vascular models	MALAT1 regulates endothelial-cell proliferation and vessel growth.
Liu, 2016 [111]	H19 and hypertrophy	Cardiomyocyte and mouse hypertrophy models	H19 modulates pathological cardiac hypertrophy through a stress-responsive mechanism.

## Data Availability

No new data were created or analyzed in this review. Data sharing is not applicable to this article.
